# Evaluating waste based retrofit strategies for thermal performance improvement in North Sinai social housing

**DOI:** 10.1038/s41598-026-64729-9

**Published:** 2026-08-12

**Authors:** Yasmin Eid

**Affiliations:** Architectural Engineering Department, Faculty of Engineering, Sinai University, El Arish, North Sinai Egypt

**Keywords:** Thermal retrofitting, Energy efficiency, Residential decarbonization, Compressed earth brick, Bio-composites, Barley straw, Date palm waste, Hot-humid climate, Egypt, Climate sciences, Energy science and technology, Engineering, Environmental sciences, Materials science

## Abstract

This study investigates thermal retrofitting strategies for single-family social housing in North Sinai, Egypt, with potential relevance to national decarbonization objectives. The research evaluates the thermal performance of locally sourced by-product materials, including barley straw (BS) and date palm (DP) waste, incorporated into compressed earth masonry and prefabricated cladding systems. These waste-based alternatives are benchmarked against the Egyptian residential building energy efficiency (RBEE) code and conventional synthetic insulation. Dynamic thermal simulations were conducted for the hot-humid climate of Bir El-Abd under both natural ventilation (NV) and mixed-mode ventilation (MMV) conditions. Simulation results indicate that the proposed interventions improved thermal performance relative to the baseline model. Under NV, the 15 cm BS masonry reduced annual discomfort hours by 23.7%, achieving approximately 89% of the discomfort reduction observed in the 60 cm theoretical variant. Under MMV, this configuration reduced annual cooling electricity use by 27.9%, with operational CO_2_ emissions decreasing to approximately 939.3 kg. Compared with the 15 cm BS masonry, the 6 cm DPM panel achieved a 21.9% reduction in discomfort hours and a 28.8% reduction in annual total heating and cooling electricity use for the South orientation. While BS masonry demonstrated higher thermal performance, the DPM cladding may offer a lower-profile alternative with reduced spatial impact. These results suggest that localized biocomposites may offer a potential pathway for reducing energy use and operational emissions in similar residential retrofit contexts.

**Supplementary Information:**

The online version contains supplementary material available at 10.1038/s41598-026-64729-9.

## Introduction

Climate change, driven by substantial greenhouse gas (GHG) emissions since the Industrial Revolution, has emerged as one of the most significant global challenges. Human activities, particularly fossil fuel combustion and unsustainable land use, have trapped solar heat within the atmosphere, leading to a steady rise in global temperatures^1,2^. Within this context, the building industry has emerged as a major contributor, with global construction increasing by 31% since 2010, 80% of which is residential. Currently, buildings account for approximately 34% of global energy-related emissions, with the residential sector alone accounting for 25% of total global energy, making their decarbonization a critical milestone toward achieving global Net-Zero goals by 2050^3,4^.

The increasing demand for space cooling significantly contributes to higher energy intensity, with space cooling now representing 20% of global electricity consumption. Since 1990, cooling demand has tripled, rising by more than 4% in 2022 due to severe heatwaves^4^. Although equipment efficiency has improved, indirect CO_2_ emissions from space cooling exceeded 1 Gt in 2022, underscoring the urgent need for passive building envelope strategies to reduce dependence on mechanical cooling^5^.

Egypt represents a relevant case within this broader energy challenge, where the residential sector accounts for approximately 37.2% of national electricity consumption^6^, according to the Egyptian Electricity Holding Company. This energy pressure is expected to intensify, as cooling demand is projected to increase by 125% by 2030^7^. This demand is particularly pronounced due to the rapid expansion of social and economic housing, which comprises nearly 40% of urban dwellings. Most of these units are constructed using traditional reinforced concrete and brick wall assemblies that lack sufficient thermal insulation, resulting in low thermal resistance and high heat transfer^8,9^.

The “fabric-first” approach is widely adopted as a retrofitting strategy that prioritizes improving the thermal performance of building envelope components, such as walls, roofs, and windows^9,10^. Insulating the building envelope can help reduce heat gain from solar radiation, which is a significant concern in Egypt’s climate^11^. External insulation can mitigate thermal bridges, while internal insulation, which occupants may install based on their financial capacity, can provide a practical, space-efficient option for improving comfort in hot climates^8,12^. Promoting circular economy principles through waste valorization aligns with this strategy by transforming local waste by-products into insulation materials, potentially offering a lower-carbon approach to upgrading existing building envelopes. Thermal conductivity (*k*) is a key indicator of insulation performance, with effective materials generally exhibiting values below 0.06 W/m·K^13^. Nevertheless, synthetic materials account for 52% of the market, raising concerns regarding health risks, substantial energy consumption during manufacturing, and limited biodegradability^9^. In regions such as North Sinai, these challenges are compounded by intense solar radiation, which can reach 28.8 MJ/m²/day during summer^14^. For low-income and Bedouin populations living in remote areas across North Sinai, where electricity has historically been supplied by diesel generators because of limited grid connectivity^15^, restricted access to mechanical cooling^16^ and the high cost of synthetic insulation^9^ may further intensify thermal stress.

Although extensive research exists, low-income housing in arid regions such as North Sinai remains insufficiently examined. This study evaluates the performance of affordable, locally sourced waste-based materials, including barley straw and date palm waste, in occupant-led retrofitting strategies. By integrating traditional housing requirements with contemporary energy efficiency measures, the research aims to inform a scalable framework for sustainable development in resource-constrained desert communities.

## Literature review

The building envelope plays a critical role in determining energy demand, with thermal insulation and airtightness contributing to improved efficiency. Heat transfer occurs via conduction, convection, and radiation, with a substantial share of thermal losses occurring through roofs (30–35%) and walls (25–30%), followed by ventilation and glazing^17^. In Egypt, several simulation-based studies have assessed retrofitting residential buildings across diverse climatic zones.

Recent research has prioritized optimizing the thermal performance of middle-income housing. For instance, the application of 70 mm extruded polystyrene (XPS) on external walls in Cairo resulted in a 33% reduction in discomfort and a 9.27% decrease in energy demand^18^. However, this analysis was limited to a single zone and did not account for roof insulation or economic feasibility. In a similar context, adherence to the Egyptian Code (RBEE) in Cairo’s hot-arid climate, utilizing 40 mm expanded polystyrene (EPS) for walls and 80 mm for roofs, achieved a 50.53% reduction in cooling load^19^. Despite these improvements, the adoption of synthetic materials is frequently constrained by high costs and energy-intensive manufacturing processes^9,17^. These limitations underscore the need for low-cost alternatives that can be integrated within structural elements.

Compressed earth bricks (CE-bricks) represent a potentially viable and cost-effective alternative^20,21^. CE-brick production costs are lower than traditional cement and clay brick by approximately 8.56% and 41.52%, respectively^20^. These unfired masonry units, composed of compacted soil and stabilizers, align with circular economy principles. Further valorization of waste by-products can improve envelope thermal performance in arid regions by reducing density and thermal conductivity while maintaining structural integrity^22^.

Hany et al.^23^ evaluated compressed earth brick (CE-brick) stabilized with alkali-activated ground granulated blast furnace slag (S. CE-brick) as a sustainable alternative to Portland cement. Their simulations indicated substantial reductions in both energy consumption and CO_2_ emissions. Among the modified slag-stabilized configurations, the incorporation of polystyrene foam (S. PF) achieved the lowest thermal conductivity (*k* = 0.698 W/m·K), whereas the standard S. CE-brick demonstrated a favorable balance between mechanical strength and thermal performance. Additionally, applying a 30 mm expanded polystyrene (EPS) insulation board to walls reduced total energy loads by 21.5% in Cairo. The present study utilizes slag due to North Sinai’s proximity to the Suez Canal industrial zone^23^, thereby repurposing industrial waste to enhance mechanical properties in Bir El-Abd.

Envelope performance enhancement depends on physical reinforcement using agricultural residues. Barley straw (BS) is considered a promising bio-aggregate due to its low thermal conductivity (*k* = 0.155 W/m·K)^22^. Natural fibers such as date palm fiber (DPF) substantially influence thermomechanical properties^24^; for example, increasing fiber length to 35 mm results in the lowest reported thermal conductivity (*k*) of 0.73 W/m·K^25^. Furthermore, date palm ash (DPA) provides a 47% reduction in thermal conductivity compared to conventional bricks, while also reducing costs by 11% and energy consumption by 7.6%^26^. Maximizing thermal efficiency can be achieved by integrating advanced finishing and insulation techniques. Fiber-reinforced mortar plasters form porous biomatrices that show improved thermal performance compared to traditional cement-based mortar^27^, and prefabricated cladding using date palm midribs (DPM) complies with RBEE standards^9^.

Although previous literature provides a useful foundation for understanding individual material performance, as summarized in Table 1, a notable gap remains in integrated research linking local waste streams with dynamic digital modeling within the specific geological and climatic context of North Sinai. The present study contributes to this area by comparatively evaluating various locally sourced additives within a simulation-based framework. The high density of palm groves, expanding barley cultivation^28,29^, and the distinctive mineralogical composition of Bir El-Abd soils, which are rich in Calcite (CaCO_3_) and Kaolinite^30–32^, provide a suitable basis for compressed earth stabilization. Rather than focusing solely on masonry unit testing, this research applies a simulation-based adaptive retrofit approach to assess envelope interventions using locally sourced waste-based additives. Furthermore, by integrating circular economy principles with performance-based assessment, the study offers a context-sensitive analytical approach for exploring thermal performance improvements in social housing, with potential relevance to comparable Mediterranean climates.

**Table 1:** Summary of previous studies on alternative materials for building envelope thermal performance improvement.

Climate/ context	Bld. typology	Key findings	Material	Thermal properties	Component	Reference
Cairo	Residential (Multifamily)	9.27% energy demand reduction; 33% discomfort reduction	XPS	*k* = 0.027–0.033 W/m·K	70 mm in walls	^18^
Cairo	Residential (Multifamily)	50.53% reduction in cooling loads under Code-compliant conditions	EPS	*k* = 0.0343 W/m·K	40 mm walls/ 80 mm roof	^19^
-	-	41.52% cost reduction compared to clay brick and 8.56% compared to cement brick	CE-brick	-	Load bearing walls	^20^
Cairo	Office	Reduction of total energy consumption and CO_2_ emissions compared to cement-stabilized CEB walls	SPF. CE-brick	*k* = 0.698 W/m·K	walls	^33^
Warm climate	-	High thermal insulation potential bio-aggregate	BS. CE-brick	*k* = 0.155 W/m·K	Walls	^22^
Algeria	-	The incorporation of 2% fibers (40 mm length) reduced the thermal conductivity by 41% compared with the reference plaster	DP. Plaster mortar	*k* = 0.396 W/m·K	Walls	^27^
Morocco	-	Incorporating 5% fibers with a length of 35 mm reduced thermal conductivity	DPF. CE-brick	*k* = 0.73 W/m·K	Walls	^25^
Dhahran, Saudi Arabia	Office	46.4% reduction in thermal conductivity, 7.6% reduction in annual energy consumption, and 11% reduction in manufacturing costs	DPA. CE-brick	*k* = 0.434 W/m·K	0.20 m /Walls	^26^
Cairo, Egypt	Residential (Single-family)	13% cooling and heating loads reduction and 4% total energy consumption saving	Insulation DPM. Pan.	Core (*k*) = 0.0496 W/m·K Cladding (*k)* = 0.1255 W/m·K	60 mm internal Walls	^9^

## Climate characteristics of the study area: North Sinai, Egypt

Egypt experiences some of the highest solar radiation levels worldwide, with an average of 3,000 sunshine hours per year^34^]. Daily sunshine duration ranges from 9 to 10 h in winter to 13 to 14 h in summer, substantially higher than that reported for many European regions^14^. North Sinai exhibits considerable seasonal variation, with solar radiation reaching 8.0 kWh/m^2^/day in summer and decreasing to 2.9 kWh/m^2^/day in winter^15^.

The 2023 Solar Radiation Atlas reports that El Arish, representing North Sinai, receives a global tilted irradiation of approximately 6.2 kWh/m^2^/day. This substantial solar resource is accompanied by an average of 8.9 sunshine hours per day, a relative humidity of 70.80%, and relatively low wind speeds averaging 2.30 m/s^34^. Although solar energy represents a potential sustainable alternative to diesel in remote regions such as Al-Arish^15^, its adoption is constrained by high initial installation costs^14^.

The climatic profile of North Sinai exhibits climatic variation between the Mediterranean coastal zone and the arid interior. Under the RBEE climatic classification, Mediterranean coastal areas in Egypt are designated as the North Coast region, whereas inland areas are assigned to different climatic categories^35,36^. Similarly, under the Köppen–Geiger classification, the North Sinai coastline, including El Arish and Bir El-Abd, is categorized as a hot semi-arid steppe climate (BSh), while much of inland Egypt falls within the hot desert climate (BWh). For simulation purposes, the TMYx weather dataset for Bir El-Abd corresponds to ASHRAE Climate Zone 2 A (Hot-Humid)^37,38^.

Along the coastal strip of North Sinai, improving the thermal performance of the building envelope represents an important design priority rather than solely an architectural preference. Under these climatic conditions, traditional passive strategies, such as natural ventilation, may be insufficient when used alone. As dependence on mechanical cooling increases, high-performance envelopes become increasingly important for improving energy efficiency and thermal comfort, particularly in low-cost housing^16^.

### Case study selection: fisherman housing in El Talul, Bir El-Abd

**Fig. 1:**
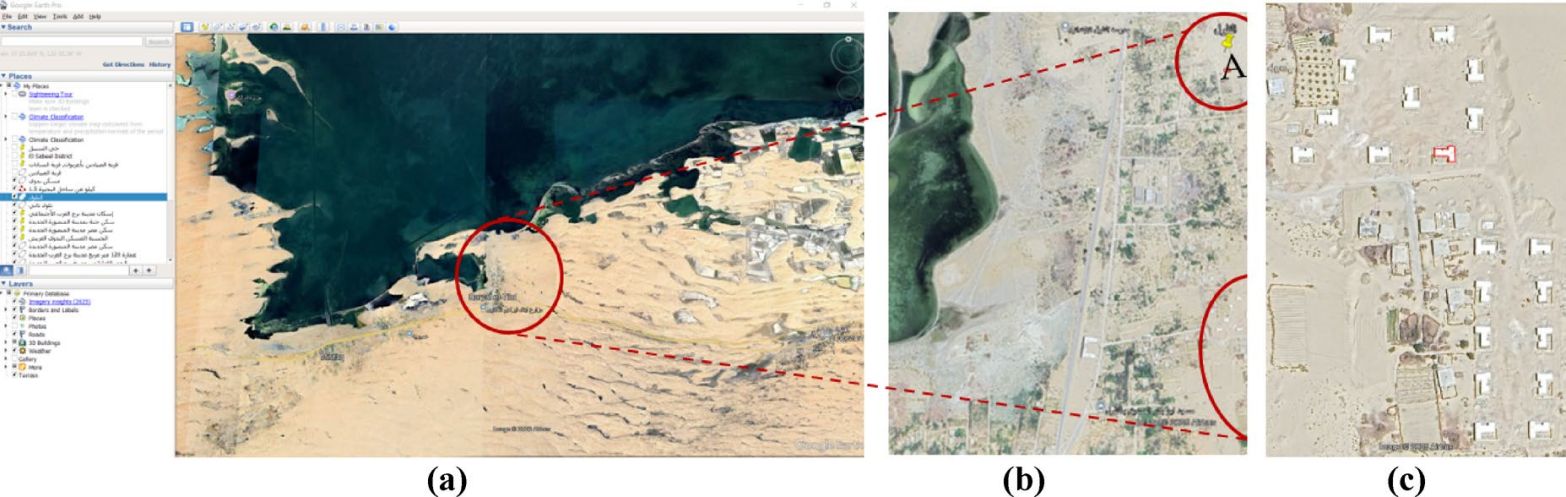
Geographic location of El Talul Village, Bir El-Abd, North Sinai: (**a**) site within the surrounding coastal context; (**b**) selected Bedouin housing zones (**A** and **B**); and (**c**) detailed view of the southern zone (**B**), illustrating the housing model orientations. Source: Prepared by the authors using Google Earth Pro^41^.

The housing model examined, locally referred to as Bedouin Housing, is part of the Fisherman Housing initiative implemented in El Sadat and El Talul villages in Bir El-Abd^39^. Units located in the El Sabihat neighborhood, approximately 500–700 m from the Lake Bardawil coastline (Fig. 1), were selected as the representative housing prototype for this study. This typology is replicated across the four cardinal orientations within the original residential development, providing a consistent basis for comparative simulation analysis. Architectural parameters were cross-checked using local media sources and regional documentation to enhance representational accuracy^39,40^.

**Fig. 2:**
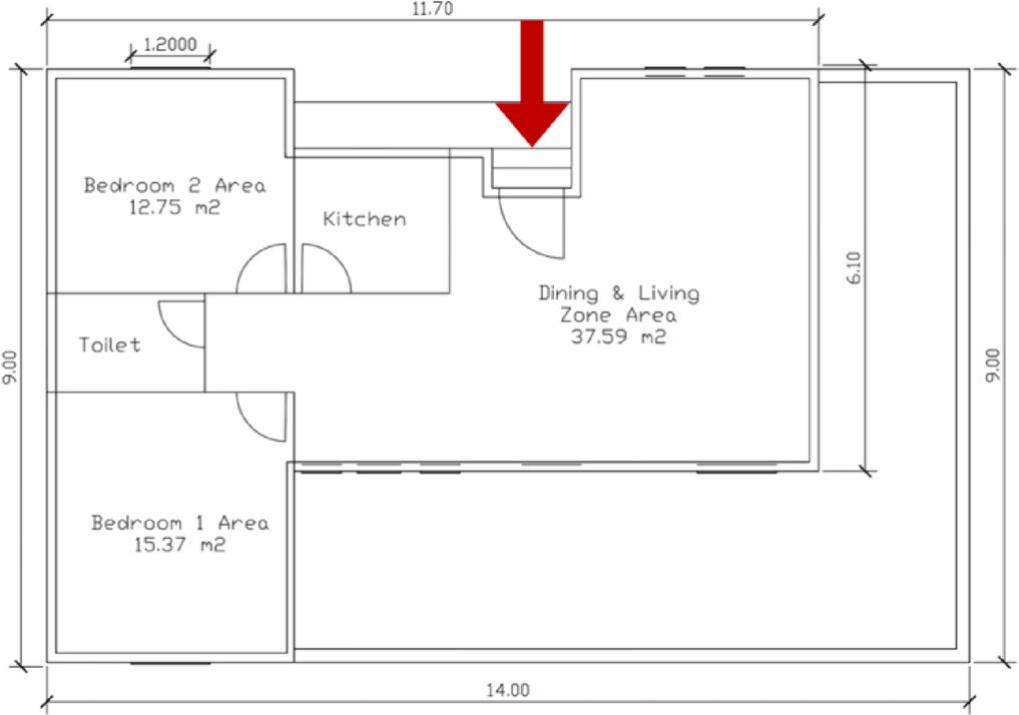
Architectural floor plan of the base-case model, indicating the reference facade orientation used in the simulation analysis.

Figure 2 illustrates a detached single-story house situated on a rectangular plot of approximately 120 m². The architectural layout adopts an L-shaped configuration with a total built-up area of 80 m², comprising a living area, kitchen, two bedrooms, and a bathroom, as well as a 40 m^2^ private backyard^39,40^. The maximum envelope dimensions are 11.7 × 9 m, resulting in a compact aspect ratio of approximately 1:1.3. This standalone configuration supports a controlled evaluation of thermal performance.

The methodological justification for selecting a standalone unit is based on the low construction density and the availability of private setbacks for outward envelope expansion. This approach helps reduce the influence of external factors, such as urban heat-island effects and complex volumetric interactions, allowing a clearer assessment of material performance and orientation effects. As a result, the observed thermal variations can be primarily interpreted in terms of material properties and orientation, supporting material selection decisions between space-efficient thin assemblies and high-mass systems. Although the analysis focuses on North Sinai, the findings may offer relevant insights for similar residential typologies in comparable hot-humid coastal contexts.

Alongside technical considerations, this social housing typology was selected for its potential vulnerability among its occupants. Low-income residents, including members of the local fishing community, may experience elevated thermal stress due to limited access to mechanical cooling options^10,42^. As occupants spend approximately 90% of their time indoors, thermal comfort becomes an important factor influencing health, well-being, and daily productivity^19,35^, highlighting the relevance of envelope-based interventions alongside traditional natural ventilation strategies.

## Methodology

**Fig. 3:**
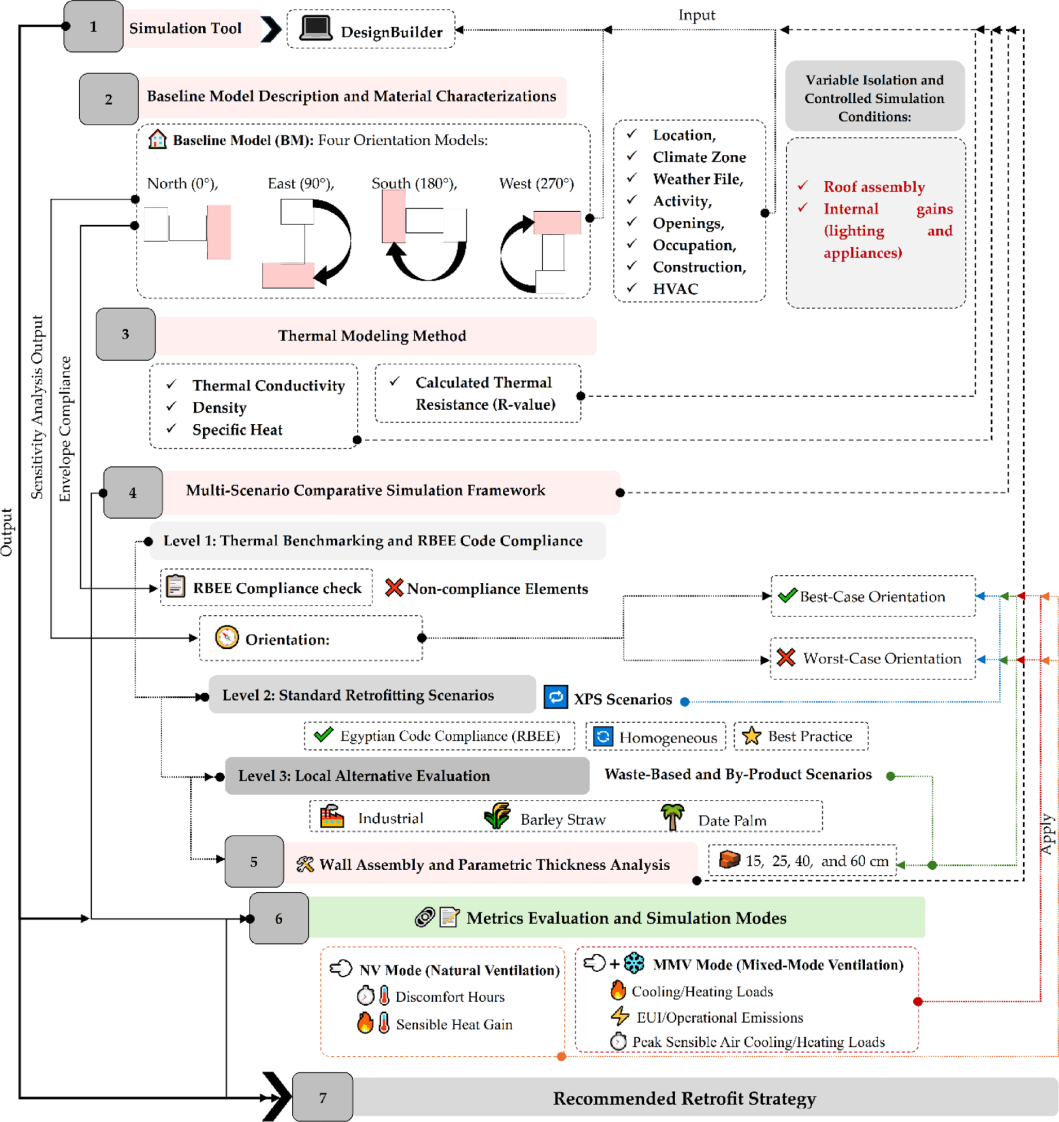
Simulation framework and methodological workflow illustrating the integration of material thermophysical properties, multi-level intervention scenarios, and thermal performance evaluation under both ventilation modes (NV and MMV).

This study integrates dynamic energy modeling (DEM) with comparative analysis to assess the thermal performance of a low-cost housing typology in a hot-humid coastal context. By benchmarking the baseline housing configuration against the RBEE Code, the research evaluates the comparative performance of conventional and locally sourced retrofit wall systems. An integrated research framework (Fig. 3) was developed to assess the thermal, energy, and operational carbon performance of alternative wall systems within the specific context of North Sinai.

### Simulation tool selection

DesignBuilder (DB) (v7.3.1.3)^43^ was selected for its capability to simulate building energy consumption and thermal behavior using the EnergyPlus dynamic engine. Its ability to model dynamic thermal behavior and natural ventilation made it suitable for implementing the research methodology and assessing the relative effects of material interventions.

### Baseline model description and material characterization

The geometric characteristics and spatial layout of the case study, as detailed in Sect. 3.1, served as the foundation for the simulation model. The construction system reflects a common Egyptian residential typology consisting of a reinforced concrete structural frame with single-layer brick infill walls^19,44^. For this study, a baseline wall configuration (U-value = 1.746 W/m^2^·K) and roof assembly (U-value = 2.002 W/m^2^·K) were selected based on established simulation-based studies and DesignBuilder material libraries^45^.

These values provide a standardized reference condition representing a moderate level of thermal performance, consistent with ranges reported in Egyptian literature (typically 1.65–2.50 W/m^2^·K for walls)^16,19,44^. In the absence of onsite experimental measurements, this baseline was adopted to establish a controlled comparative framework. Consequently, the analysis focuses on the relative performance of retrofit scenarios using waste-based materials compared with a consistent reference case, rather than the absolute prediction of real-world building performance.

Table 2 summarizes the thermophysical properties of common masonry materials available in the Egyptian market. Reported thermal conductivity (*k*) values range from 0.33 to 1.6 W/m·K. The selected baseline value (*k* = 0.72 W/m·K) lies near the midpoint of this range, thereby reducing potential bias toward extreme performance conditions and supporting a balanced comparative evaluation of the proposed material alternatives.

**Table 2:** Thermophysical properties of representative masonry materials available in the Egyptian market^36,46^ and the baseline reference case.

Material type	Hollow leka block	Solid red brick	Hollow red brick	Solid cement brick A	Solid cement brick B	Hollow cement brick	Limestone brick	Sand brick	Hollow sand brick	Reference case^45^
Thermal conductivity, *k* (W/m·K)	0.39	1.00	0.60	1.25	1.4	1.6	0.33	1.59	1.39	0.72
Density (kg/m^3^)	1200	1950	1790	1800	2000	1140	985	1800	1500	1920
Specific heat capacity (J/kg·K)	1000	829	840	880	840	880	850	835	811	840

**Fig. 4:**
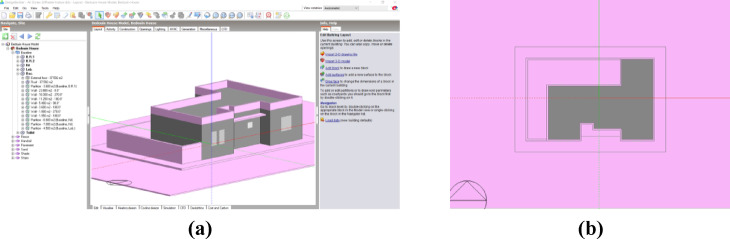
Three-dimensional simulation model of the selected Bedouin house developed in DesignBuilder: (**a**) perspective view and (**b**) top view illustrating the baseline model orientation. Source: prepared by the author using DesignBuilder software.

A detailed DEM was constructed in DesignBuilder (DB) to establish the baseline model (BM), representing the case study’s as-is condition (Fig. 4). The simulation incorporated the case-study geometric data and the previously described material properties across all four cardinal orientations. To ensure consistency in labeling simulation scenarios (Table 3), building orientation was defined as the direction of the longest facade (the entrance side).

The simulation employed a site-specific Typical Meteorological Year (extended) (TMYx) weather file for Bir El-Abd (2004–2016)^37^. Geographical parameters, including latitude, longitude, and elevation, were derived directly from the embedded metadata within the TMYx file and linked to the DB. This setup ensured consistency between the climatic dataset and the associated solar geometry and shading calculations, eliminating the need for manual coordinate input and supporting representative simulation of ASHRAE Climate Zone 2 A conditions.

**Table 3:** Building orientation scenarios defined by the direction of the longest facade (the entrance side).

			
North orientation (0°)	East orientation (90°)	South orientation (180°)	West orientation (270°)

Table 4 presents the simulation input parameters used for dynamic thermal simulations. These parameters include the weather dataset, the climatic classification, and the operational schedules, adapted to reflect the local context. The envelope data include the thermophysical properties of the baseline building components applied across the four orientation-based baseline simulation models, as described in Sect.  4.2. To reflect the household characteristics of the selected case study context, occupancy density and activity levels were modeled based on the typical structure of a fisherman’s household. Furthermore, operational parameters such as natural ventilation schedules, HVAC setpoints, and seasonal adjustments are provided to support reproducibility of the simulation framework.

**Table 4:** Input parameters and simulation settings used in DesignBuilder (DB).

Location	Site location:		Latitude: 30.97°; Longitude: 32.77 °^37^					
	ASHRAE climate zone		2 A					
	Elevation above sea level (m)		24					
	Hourly Weather Data (EPW)		Bir El-Abd, using the EPW file “EGY_SS_Bear.El.Abd.624540 TMYx (2004–2016)” [37].					
	Include Fuel Emissions		Electricity					

	Zone		Reception & dining zone		Bedroom 1			Bedroom 2
Activity	Area (m^2^)		37.59		15.37			12.75
	Occupancy Density (people/m^2^)		(Family of five) 0.1330		(Three children) 0.195			(Master bedroom) 0.157
	Zone Height (m)		3.00					
	Heating Setpoint (℃)		20.0					
	Heating Setback (℃)		15.0					
	Cooling Setpoint (℃)		27.0					
	Cooling Setback (℃)		31.0					
	Limit Type:		Limit flow rate and capacity					
	Environmental Control		Indoor minimum temperature control (25.0 ℃, fixed by value)					
Openings	Doors		Wooden door					
	Frame and Glazing		50 mm wooden frame with 3 mm single clear glazing, external wooden solar louvers, and 85% operable glazing area.					
	Window-to-Wall Ratio (WWR)		North Wall 15%		South Wall 13%			North Wall 13%
			South Wall 12%					
Occupancy schedule	Reception and dining zone (Rec.)							
	Bedrooms 1 and 2 (BR1 and BR2)							
	Note: Blue indicates occupied hours; beige indicates unoccupied hours.							
Construction specifications adapted from Raslan et al. 2025 [45]	Thermal properties of baseline building components modeled in DesignBuilder							
	Roof Thickness = 0.27 m R-value = 0.500 m^2^·K/W U-value = 2.002 W/m^2^·K			Floor Thickness = 0.29 m R-value = 0.448 m^2^·K/W U-value = 2.234 W/m^2^·K				
	Internal Walls Thickness = 0.165 m R-value = 0.458 m^2^·K/W U-value = 2.18 W/m^2^·K			External Walls Thickness = 0.29 m R-value = 0.573 m^2^·K/W U-value = 1.746 W/m^2^·K				
HVAC	Natural Ventilation (NV)	Natural ventilation mode with no active heating or cooling; scheduled ventilation defined using DesignBuilder template slider airtightness settings.						
	Mixed Mode Ventilation (MMV)	Split HVAC system with grid-supplied electricity and no fresh air input; heating and cooling operated according to occupancy schedules, with natural ventilation enabled.						

Electrical appliances, including computers, equipment, general lighting, and domestic hot water (DHW), were disabled during both simulation modes.

#### Variable isolation and controlled simulation conditions

To ensure that observed variations in thermal comfort and energy demand are solely attributable to the proposed wall material interventions, rigorous control of confounding variables was implemented. The roof assembly was maintained as a constant, code-compliant element across all scenarios. This approach prevents the disproportionate heat gain typically associated with non-insulated roofs in detached houses from obscuring the performance differences of waste-based wall materials. Internal heat gains from artificial lighting, domestic hot water (DHW) systems, and electrical equipment, including computers and general appliances, were excluded. Maintaining these sources in an off state establishes a normalized simulation environment, isolating envelope-driven heat transfer from variability introduced by internal equipment use^47^. All other boundary conditions, including infiltration rates and HVAC setpoints (20–27 °C), were kept identical across the baseline, industrial, and by-product-based models. This ceteris paribus approach ensures that the thermo-physical properties of the materials remain the sole independent variable under investigation.

### Thermal modeling approach

To ensure consistency across all simulation levels, the thermal properties of both commercial and waste-by-product materials were incorporated into the DB, including thermal conductivity (W/m·K), specific heat (J/kg·K), and density (kg/m^3^). When comprehensive physical data for local materials were unavailable in the literature, the thermal resistance (*R-value*) approach was adopted. The R-value for each material layer was manually calculated based on its thermal conductivity (*k*) and assigned thickness (*T*), as shown in Eq. (1)^9^:

1 $$\:\boldsymbol{R}=\frac{\boldsymbol{T}}{\boldsymbol{k}}$$

where:

#### R

the thermal resistance (m^2^·K/W).

#### T

the thickness of the material (m).

#### k

the thermal conductivity (W/m·K).

This approach was applied to all examined materials at Levels 2 and 3, except for date palm plaster and date palm ash masonry. This ensured a standardized benchmark for comparing the thermal performance of the proposed interventions.

### Multi-scenario comparative simulation framework

The simulation framework is organized into three sequential levels to assess the building’s thermal evolution and potential for performance improvement. The methodology advances through the following levels:

#### Level 1: thermal benchmarking and RBEE code-compliance

A sensitivity analysis was conducted to evaluate thermal performance across the four principal building orientations defined in Table 3. Based on the results, two representative orientations were selected for the subsequent evaluation levels to capture the range of thermal responses under contrasting solar exposure conditions. The first represents the “best-case” orientation, in which the longest facades (11.7 m) face north and south (i.e., the building’s longest axis is oriented east-west), consistent with Egyptian passive design recommendations for minimizing solar gains from the east and west^36^. The second represents the “worst-case” orientation, in which the longest facades face east and west (i.e., the building’s longest axis is oriented north-south), thereby maximizing solar exposure. Including this unfavorable orientation provides a conservative assessment of the proposed retrofit strategies under the most thermally demanding conditions.

This comparative approach evaluates the proposed materials across the building’s thermal spectrum, with particular attention to RBEE Code requirements for facades exposed to high solar loads. Following the Variable Isolation protocol (Sect. 4.2.1), the study isolates the impact of the wall assembly by maintaining a constant, code-compliant roof R-value. This methodology supports the objective of developing scalable solutions for the broader residential sector. Although roofs contribute 30–35% of heat transfer in standalone units^17^, their influence is reduced in multi-story buildings. By neutralizing the roof’s thermal load, the simulation ensures that incremental performance gains from wall interventions are accurately measured. The Level 1 baseline was therefore assessed against these standards (Table 5) to identify existing thermal deficiencies.

**Table 5:** Thermal performance criteria for opaque and transparent building envelope elements in the North Coast Region of Egypt^35^.

Component orientation		*R*-value (m^2^·K/W)		SHGC ^*^ limits by WWR (%)				SGR^**^ requirements by WWR^***^ (%)			
		Unconditioned	Conditioned	< 10	< 20	< 30	> 30	< 10	< 20	< 30	> 30
Roof		2.15–2.45	2.5	-							
Walls	North	0.35–0.47	0.50–0.62	-	-	-	0.71	-	-	-	60%
				-	-	-	0.71	-	-	-	60%
	East/West	0.72–1.15	0.87–1.30	0.65	0.5	-	-	60%	70%	-	-
				0.65	0.5	0.4	0.35	60%	70%	80%	90%
	South	0.47–0.69	0.62–0.84	-	0.71	0.64	0.55	-	60%	70%	90%
				-	0.71	0.64	0.55	-	60%	70%	90%

*SHGC: For unshaded openings, SHGC must remain below the maximum regulated threshold to limit solar heat gain. When external solar shading devices (e.g., wooden louvers or movable shading screens) are integrated, SHGC requirements are not mandatory.

**SGR: The shaded glass ratio must meet or exceed the minimum specified threshold to ensure adequate solar protection.

***WWR: Window-to-Wall Ratio

Intercardinal orientations (e.g., Northeast and Southwest) were excluded from the analysis, as the building model was aligned with the four cardinal orientations.

#### Level 2: standard retrofitting scenarios (XPS intervention)

Level 2 examines standard commercial insulation by employing extruded polystyrene (XPS) as a benchmark. This material was selected based on recommendations from previous studies for use in hot regions^19^ and on its demonstrated effectiveness in simulated assessments^18^. An Egyptian-manufactured XPS product (*k* = 0.028 W/m·K)^48^ was modeled in three strategic scenarios. In each scenario, XPS is applied as an external insulation layer, a common practice in building retrofitting to improve thermal resistance without reducing the internal floor area. These scenarios are designed to address the performance gaps identified in Level 1 and ensure compliance with mandatory RBEE standards (Tables 6 and 7). To systematically evaluate these improvements, the following scenarios evaluate different insulation thicknesses and placement strategies, as outlined below:

- **Scenario A: RBEE code compliance (X-C)**.

In Scenario A, insulation is selectively applied to opaque envelope components that fall below the RBEE Code thermal resistance requirements identified in Level 1. The insulation thickness was determined to achieve code-compliant total R-values for the relevant envelope components under both unconditioned and conditioned building standards. The resulting effects on thermal comfort and energy efficiency are then assessed.

- **Scenario B: homogeneous walls assembly configuration (X-H)**.

Building upon the previous phase, Scenario B adopts a holistic approach. Roof insulation remains unchanged, while wall insulation is applied uniformly in all orientations at a 20 mm XPS thickness. This approach exceeds minimum code standards to ensure a homogeneous assembly and consistent thermal resistance around the building perimeter.

- **Scenario C: best practice configuration (X-BP)**.

In the final phase, insulation thickness is evaluated to improve energy performance and occupant comfort. Based on research recommendations, a standardized 60 mm XPS layer is applied across the entire building envelope^18,49^. This best practice strategy advances the building beyond basic compliance toward optimal efficiency.

These scenarios establish a “performance ceiling” for Level 2. Instead of conducting a comprehensive market survey, the aim is to define a high-performance commercial benchmark for evaluating affordable, local bio-based alternatives at Level 3.

**Table 6:** Summary of level 2 XPS intervention scenarios.

Scenarios	Description	Target	Thickness of XPS (mm)	
			Wall	Roof
A. X-C	Standard code compliance	Compliance with local regulatory standards^*^	20 ^*^	60
B. X-H	Homogeneous wall assembly configuration	Uniform insulation distribution^**^	20 ^**^	
C. X-BP	Best practice configuration	Best-practice insulation benchmark ***	60 ^***^	

^*^Applied only to opaque envelope components that do not comply with RBEE Code thermal resistance requirements.

^**^Uniform 20 mm insulation applied to all facades (North, East, South, and West).

^***^Uniform 60 mm insulation applied to all facades and the roof.

**Table 7:** Parametric matrix for Level 2 XPS intervention scenarios.

Scenario A (X-C): standard code compliance	
Walls	Roof
Thickness = 0.31 m R-value = 1.287 m^2^·K/W	Thickness = 0.40 m R-value = 2.643 m^2^·K/W


Scenario C (X-BP): Best Practice Configuration	
Walls	Roof
Thickness = 0.35 m R-value = 2.716 m^2^·K/W	Thickness = 0.40 m R-value = 2.643 m^2^·K/W


Note: XPS R-values were calculated using Eq. (1) for DB input parameters.

#### Level 3: evaluation of local waste-based alternatives

At this stage, alternative retrofit strategies are evaluated by integrating cost-effective, locally sourced materials chosen for their abundance in North Sinai. To accurately isolate the effects of wall interventions, roof insulation was installed in accordance with the standards established for Level 2 Scenario A. This approach ensures that observed variations in thermal comfort and energy loads are attributable solely to the wall systems. The following configurations were systematically selected based on their optimal thermal performance as documented in established literature:

- **Core wall construction**: Four sustainable brick configurations, selected based on their demonstrated thermal performance in previous research, were evaluated to identify the most effective structural core.

1. CE-brick (Standard & Improved)^33^: Three configurations were evaluated: the standard CE-brick as a baseline, compressed earth with slag (S. CE-brick), and the hybrid slag with polystyrene foam (SPF. CE-brick), with the latter two identified as high-performing configurations.
2. BS. CE-brick^22^: The unfired compressed earth brick reinforced with barley straw was evaluated, with the configuration demonstrating the highest thermal efficiency (lowest conductivity) selected.
3. DPF. CE-brick^25^: A compressed earth brick reinforced with 35 mm date palm fibers (DPF) was included, representing an optimal balance between structural durability and the insulating properties of biofibers.
4. DPA. CE-brick^26^: A masonry unit incorporating date palm ash (DPA) as a partial replacement for cement was included, selected for its demonstrated capacity to reduce annual energy consumption and mitigate internal heat storage.

- **DP thermal plaster**^27^: A bio-based mortar plaster reinforced with date palm fibers (DP. Pl.) was applied in thicknesses of 20 mm and 40 mm. The plaster mortar was evaluated in both external and internal configurations to determine the optimal application strategy.
- **DPM cladding system**^9^: To enhance the building envelope, a DPM cladding system (DPM. Pan.) was integrated internally. Due to the low thermal conductivity of the DPM nonwoven panel, this layer functions as a high-performance bio-insulation alternative to synthetic materials.

### Wall assembly and parametric thickness analysis

This stage evaluates the thermal retrofitting of an existing residential envelope using local and sustainable alternatives. Each intervention was subjected to an independent parametric analysis, considering four masonry units with thicknesses of 15, 25, 40, and 60 cm, along with bio-based mortar and the 6 cm DPM panel (Table 8). The 15–25 cm range represents standard construction practice, whereas 40 and 60 cm were included as theoretical upper limits to capture the full spectrum of thermal performance and identify the point of diminishing returns. This parametric approach is consistent with established research methodologies, including those outlined by Hany et al.^33^, which examine extreme thickness ranges to define efficiency thresholds and establish standardized benchmarks for thermal lag and damping effects relative to insulation standards, such as the 6 cm XPS.

As this study focuses on post-construction retrofitting, the placement of interventions was determined by functional design requirements and the need to preserve net internal floor area. Masonry units incorporating waste by-products and external mortars were therefore modeled as external cladding layers to maximize thermal mass and protect the existing building fabric. In contrast, the date palm (DP) panel was incorporated as an internal lining layer, aligning with the intended function of enhancing localized thermal comfort and facilitating installation in existing spaces. This hybrid strategy ensures that masonry additions extend outward, while specialized interior panels provide thermal damping without altering the existing spatial configuration.

**Table 8:** Material thermal properties, proposed wall assembly configurations, and resulting whole-wall R-values.

	CE-brick: thermal conductivity (k) = 1.262 W/m·K^33^					
CE-Brick Industrial By-Product Interventions	Material thickness (m)					
	0.15	0.25		0.40		0.60
	Calculated material *R*-value (m²·K/W)					
	0.119	0.198		0.317		0.475
	Wall thickness (m)					
	0.44 m	0.54 m		0.69 m		0.89 m
	Whole-wall *R*-value (m²·K/W)					
	0.692	0.771		0.890		1.048
			
	S. CE-brick: thermal conductivity (k) = 0.977 W/m·K^33^					
	Material thickness (m)					
	0.15	0.25		0.40		0.60
	Calculated material *R*-value (m²·K/W)					
	0.154	0.2559		0.409		0.6141
	Wall thickness (m)					
	0.44	0.54		0.69		0.89
	Whole-wall *R*-value (m²·K/W)					
	0.726	0.829		0.982		1.187
			
	SPF. CE-brick: thermal conductivity (k) = 0.698 W/m·K^33^					
	Material thickness (m)					
	0.15	0.25		0.40		0.60
	Calculated material *R*-value (m²·K/W)					
	0.215	0.358		0.573		0.859
	Wall thickness (m)					
	0.44	0.54		0.69		0.89
	Whole-wall *R*-value (m²·K/W)					
	0.788	0.931		1.146		1.432
			
BS. CE-Brick Interventions	BS. CE-brick: thermal conductivity (*k)* = 0.155 W/m·K^22^					
	Material thickness (m)					
	0.15	0.25		0.40		0.60
	Calculated material R-value (m²·K/W)					
	0.9677	1.6129		2.5806		3.8709
	Wall thickness (m)					
	0.44	0.54		0.69		0.89
	Whole-wall R-value (m²·K/W)					
	1.554	2.186		3.153		4.444
			
	*DP. Pl.: thermal conductivity *(k)* = 0.396 W/m·K, Specific heat = 2088.82 J/kg·K, and Density = 756.48 kg/m^3^^27^					
	Material thickness (m)					
	0.02			0.04		
	Wall thickness (m)					
	0.31			0.33		
	Whole-wall R-value (m²·K/W)					
	0.623			0.674		
			
	*DPA. CE-brick: thermal conductivity *(k) =* 0.434 W/m·K, Specific heat = 2032 J/kg·K, and Density = 780 kg/m^3^^26^					
	Material thickness (m)					
	0.15	0.25		0.40		0.60
	Wall thickness (m)					
	0.44	0.54		0.69		0.89
	Whole-wall R-value (m²·K/W)					
	0.941	1.172		1.517		1.978
			
DP Interventions	DPF. CE-brick: Thermal conductivity (*k)* = 0.73 W/m·K^25^					
	Material thickness (m)					
	0.15	0.25		0.40		0.60
	Calculated material R-value (m²·K/W)					
	0.2055	0.3425		0.5479		0.8219
	Wall thickness (m)					
	0.44	0.54		0.69		0.89
	Whole-wall R-value (m²·K/W)					
	0.801	0.938		1.143		1.417
			
	DPM. Pan.^9^:		Core	Cladding sheet		
	Thermal conductivity *(k)* (W/m·K):		0.0496	0.1255		
	Material thickness (m):		0.03	0.03		
	Calculated material R-value (m²·K/W):		0.6048	0.2390		
	Wall thickness (m):		0.35			
	Whole-Wall R-value (m²·K/W):		1.434			

*Only materials marked with an asterisk (*) had complete thermophysical properties and were directly entered into DB; all other materials were modeled using R-values calculated according to Eq. (1).

For DP. Pl. = Date Palm Plaster; E = external application; I = internal application; numeric values indicate material thickness in meters (0.02 and 0.04 m).

### Evaluation of metrics and simulation modes

Multiple scenarios are benchmarked under harmonized boundary conditions, including standardized occupancy, zero-equipment load protocols to isolate envelope impact, and consistent weather data to reduce computational bias. The assessment uses a dual-mode operational simulation comprising natural ventilation (NV) and mixed-mode ventilation (MMV). The NV mode represents passive conditions and focuses on discomfort hours, while the MMV approach incorporates mechanical systems that operate only when passive strategies are insufficient to maintain comfort. The performance of these modes is assessed using two critical metrics:

#### Passive mode: adaptive thermal comfort (natural ventilation)

This phase evaluates the inherent thermal performance of the building envelope, a factor essential for low-cost projects that depend on passive cooling strategies. Long-term thermal comfort performance is quantified using the percentage of discomfort hours as the primary metric. This parameter enables dynamic longitudinal analysis by incorporating seasonal variations in occupant clothing levels (Clo values), humidity, and operating temperatures, thereby overcoming the limitations of momentary peak-load assessments^43^.

The ASHRAE 55 Adaptive Comfort Model is central to this assessment and is specifically designed for naturally ventilated spaces in hot, arid climates. This model establishes a relationship between the mean hourly outdoor temperature and the indoor operating temperature. Consistent with the literature^19^, the 80% acceptability limit, defined by a ± 3.5 ℃ threshold, is used to determine the thermal comfort range. This framework incorporates physiological and behavioral adaptations, providing a realistic metric for evaluating the impact of structural interventions on perceived indoor environmental quality.

#### Active mode: energy consumption and loads (MMV)

In the MMV scenario, thermal comfort is maintained as a constant operational constraint (20–27 °C), thereby highlighting the energy penalty required to sustain these conditions. Energy use intensity (EUI), measured in kWh/m², serves to evaluate long-term economic and environmental performance. The EUI and operational CO_2_ emissions presented are used as comparative performance indicators, not as absolute predictive values for total household consumption. By excluding internal heat gains from lighting and appliances, the simulation isolates envelope-driven energy demand, ensuring that observed savings are attributable solely to material interventions. This metric quantifies electricity consumption for heating and cooling, thereby distinguishing mechanical energy demand from other building loads.

To further assess environmental impact, these energy requirements are converted into operational CO_2_ emissions, representing the building’s footprint during the usage phase. Operational CO_2_ emissions were derived directly from the DB simulation outputs. DesignBuilder calculates these emissions based on the simulated heating and cooling electricity consumption using the Generic electricity emission factor provided within the DB Fuel Emission Factors database (168.33 g/MJ, equivalent to 0.606 kg CO_2_/kWh). Since all simulation scenarios were evaluated using the same emission factor, the comparative assessment of retrofit alternatives remains unaffected. Consequently, operational CO_2_ emissions are presented as software-generated performance indicators rather than manually calculated values. This study focuses exclusively on operational emissions; embodied carbon from material extraction, manufacturing, and end-of-life phases is excluded from the current analysis. By comparing the relative energy reductions between the baseline and retrofitted scenarios, the analysis isolates the impact of material efficiency, ensuring that the findings are attributable solely to the proposed bio-based interventions.

The dual-metric approach, integrating Energy Use Intensity (EUI) and Discomfort Hours, enables a comprehensive comparison between baseline and optimized scenarios. Under natural ventilation (NV), the analysis correlates discomfort hours with sensible heat gain to identify the primary drivers of thermal stress. In contrast, the mixed-mode ventilation (MMV) assessment focuses on heating and cooling loads, peak thermal demands, and sensible heat gain, distinguishing its evaluation from the NV analysis. Together, these metrics establish a thermal performance baseline for non-insulated social housing in North Sinai and provide a consistent framework for evaluating the relative effectiveness of the proposed bio-based retrofit interventions.

## Results

### Baseline thermal performance across the cardinal orientations

#### Level 1: BM thermal benchmarking and RBEE code compliance

**Fig. 5:**
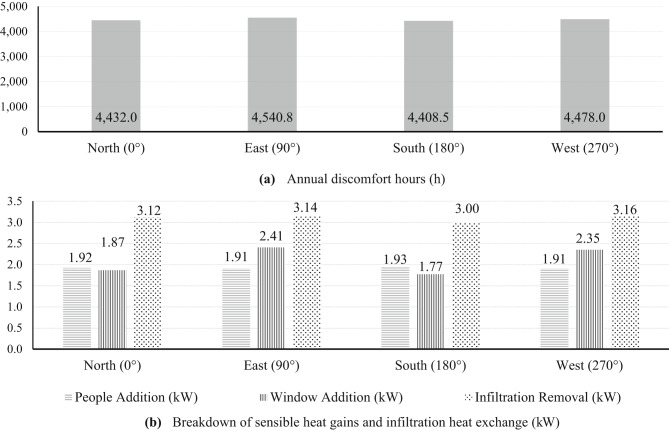
Comparative analysis of passive thermal performance for the BM across the four cardinal orientations under NV mode.

**Fig. 6:**
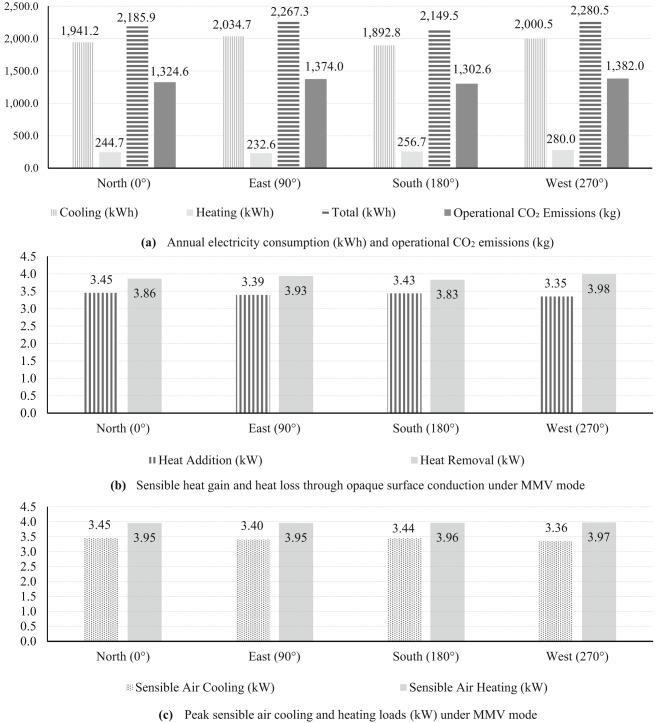
Comparative analysis of energy performance metrics for the BM across the four cardinal orientations under MMV mode.

This initial phase established a performance baseline for the BM across the four cardinal orientations under NV and MMV modes, as shown in Figs. 5 and 6.

- **NV mode and thermal drivers**: Fig. 5 shows that annual discomfort hours exceeded 4,400 h across all baseline orientations under NV conditions. The South (180°) recorded the lowest discomfort hours at 4,408.5 (50.3% of the 8,760 annual hours^19^), while the East (90°) recorded the highest at 4,540.8 h (51.8%). Although the variance in discomfort hours across orientations is only 1.5% points, the model remains outside comfort conditions for approximately half of the year. Sensible heat gain appears to be the dominant thermal contributor, peaking through glazing at 2.41 kW in the East orientation. Internal gains from occupants remained steady across all orientations, while infiltration-driven heat loads were highest in the West at 3.16 kW.
- **MMV mode and energy metrics**: Fig. 6 shows clear differences in energy performance across the four orientations. The East recorded the highest annual cooling electricity consumption (2034.7 kWh), while the West had the highest heating electricity use (280.0 kWh). Consequently, the West was the lowest-performing orientation, with a total simulated annual electricity consumption of 2,280.5 kWh and operational CO_2_ emissions of 1,382 kg. Opaque surface conduction resulted in the highest heat removal in the West orientation, reaching 3.98 kW, whereas the highest heat addition was observed in the North orientation at 3.45 kW. Peak thermal loads remained within a relatively narrow range, with sensible air heating stabilizing at approximately 3.97 kW in the West orientation and sensible air-cooling peaking at 3.45 kW in the North orientation. The South orientation exhibited the most favorable overall energy performance, yielding the lowest operational CO_2_ emissions of 1,302.6 kg.
- **Code compliance and benchmarking**: The BM did not fully meet RBEE standards, as shown in Table 9. The roof had an R-value of 0.50 m^2^·K/W. Wall assemblies recorded 0.573 m^2^·K/W, which fits North/South criteria for unconditioned buildings but does not meet the 0.72–1.15 m^2^·K/W needed for East/West facades. For conditioned buildings, all wall R-values, except those on North facades, were below the required ranges 0.87–1.30 m^2^·K/W.

**Table 9:** Comparison of simulated BM envelope performance with RBEE code requirements.

	Facade orientation		Code *R*-value (m^2^·K/W) [35]				BM (DB Output) *R*-value (m^2^·K/W)
			Unconditioned Building		Conditioned Building		
			Value	Compliance	Value	Compliance	
	Roof		2.45	✗	2.5	✗	0.50
	Wall	North	0.35–0.47	✓	0.50–0.62	✓	0.573
		East/West	0.72–1.15	✗	0.87–1.30	✗	
		South	0.47–0.69	✓	0.62–0.84	✗	

✓ = Compliant with the RBEE code requirement; ✗ = Non-compliant with the RBEE code requirement.

At Level 1, the BM analysis revealed differences in thermal performance across all orientations. The South orientation emerged as the best-case scenario, recording the lowest annual discomfort hours and operational energy demand. Conversely, the West and East orientations were identified as the worst-case scenarios; specifically, the West orientation exhibited the highest combined cooling and heating loads associated with higher solar gains. These findings establish the basis for subsequent levels, in which the South orientation is used to assess the performance of waste-based materials, while the East and West are used to test the selected configurations under more thermally demanding conditions.

#### Level 2: standard retrofitting (XPS Interventions): South orientation analysis

**Fig. 7:**
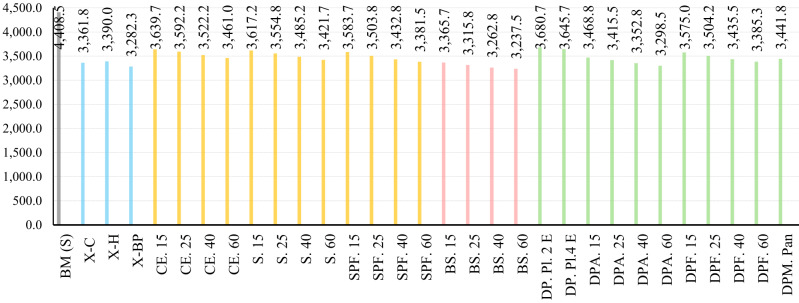
Comparison of annual discomfort hours (h) across all intervention scenarios under NV mode, relative to the BM in the South orientation. Numeric labels denote material thicknesses in centimeters.

**Fig. 8:**
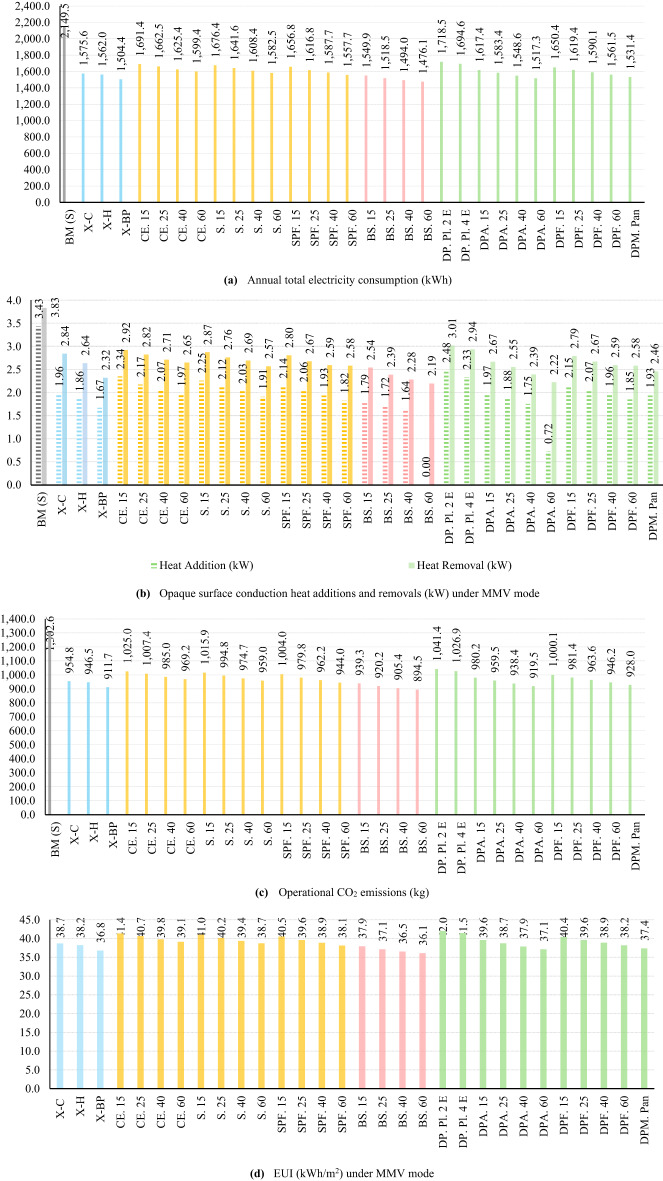
Comparative energy and thermal performance of all intervention scenarios under MMV mode, relative to the BM in the South orientation. Numeric labels denote material thicknesses in centimeters.

**Fig. 9:**
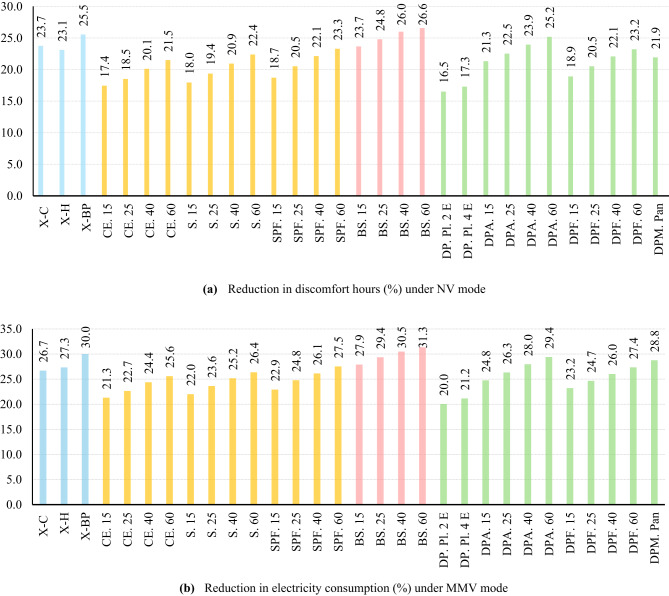
Comparative percentage improvements in thermal and energy performance of all intervention scenarios for the South orientation under NV and MMV modes. Numeric labels denote material thicknesses in centimeters.

To address the thermal deficiencies identified in the BM, three insulation levels were studied: X-C (Code Compliance), X-H (Homogeneous Wall), and X-BP (Best Practice). Based on Level 1 findings, the evaluation focused on the South orientation, chosen as the primary target for intervention and as the best-case scenario for performance analysis. To quantify the impact of insulation, simulation results shown in Figs. 7 and 8, and Fig. 9 compare these insulation levels under both NV and MMV modes. This assessment aims to identify relative improvements in thermal comfort and energy efficiency for conventional XPS insulation configurations.

- **NV mode and thermal comfort**: In NV mode, annual discomfort hours decreased from 4,408.5 in the BM to 3,361.8 in X-C and 3,390.0 in X-H, reductions of 23.7% and 23.1%, respectively. The X-BP configuration, using 60 mm of XPS insulation, achieved the best performance with 3,282.3 h, a 25.5% reduction compared with the BM and a further 1.8% points reduction in discomfort hours relative to X-C (Figs. 7 and 9a). Infiltration heat exchange also declined from 2.99 kW in BM to 2.37 kW in X-BP (Appendix A).
- **MMV mode and energy metrics**: Under MMV conditions, electricity consumption and operational emissions were substantially reduced. Annual cooling electricity consumption decreased from 1,892.8 kWh in the BM to 1,309.7 kWh in X-BP, representing a 30.8% reduction (Figs. 8a and 9b). EUI decreased from 52.4 kWh/m^2^ to 36.8 kWh/m^2^, corresponding to a 29.8% reduction. The X-C scenario achieved a result closely comparable to X-BP, although its EUI reduction remained 3.6% points lower (Fig. 8d). As a result, annual operational CO_2_ emissions in X-BP decreased by 30.0% relative to the BM, from 1,302.6 kg to 911.7 kg (Fig. 8c).
- **Envelope performance**: For envelope-specific metrics, opaque surface conduction heat gain decreased by 51.4%, from 3.43 kW to 1.67 kW in the X-BP scenario (Fig. 8b). Peak sensible air-cooling and heating loads were reduced by approximately 45.7% and 41.6%, respectively (Appendix A).

#### Level 3: evaluation of waste-based alternatives: South orientation analysis

At Level 3, the study assessed the integration of by-product-based material layers into existing wall assemblies to improve thermal performance. It also examined the effect of increasing masonry thickness from 15 cm to 60 cm, assessing the trade-off between environmental performance and practical constraints. Following the Level 1 findings, which identified the South orientation as the best-performing case, all Level 3 interventions were applied to all facades in this orientation. As shown in Figs. 7 and 8, and 9, the evaluated waste-based interventions generally improved both passive and active building performance metrics. Among the evaluated variants, the barley straw (BS) brick showed the strongest thermal performance. For finishing layers, date palm plaster (DP. Pl.) was evaluated for both internal and external applications; however, internal results were omitted from the charts for clarity, as they showed performance comparable to the corresponding external application. Accordingly, date palm plaster was retained as an external finish for subsequent analyses.

- **NV mode and thermal discomfort**: In the South orientation, simulations show that increasing wall thickness consistently reduces discomfort hours. All interventions lowered discomfort by approximately 728–1,171 h, as shown in Figs. 7 and 9a. The 15 cm BS reduced discomfort to approximately 3,365.7 h, a 23.7% reduction over the BM value of 4,408.5 h. Further increases to 25, 40, and 60 cm reduced discomfort to 3,315.8 h (24.8%), 3,262.8 h (26.0%), and 3,237.5 h (26.6%), respectively.
- **Thickness–performance relationship**: The 15 cm BS variant achieved a 23.7% reduction in discomfort hours. Increasing the thickness to 60 cm resulted in only an additional 2.9% points of improvement, indicating diminishing returns. Most of the observed thermal benefits were achieved within the 15–25 cm range, while increasing the thickness from 25 cm to 60 cm yielded only a further 1.8% points reduction in discomfort hours (Fig. 9a).
- **MMV mode and operational CO**_**2**_: The interventions under MMV mode reduced heating and cooling electricity consumption by 20–31.3% compared with the BM (Fig. 9b). The 15 cm BS configuration recorded a total electricity consumption of 1,549.9 kWh and operational CO_2_ emissions of 939.3 kg with MMV operation, serving as the reference configuration for the optimized BS masonry configurations (Fig. 8a and c). The 25 cm BS configuration reduced total electricity consumption to 1,518.5 kWh and operational CO_2_ emissions to 920.2 kg, representing a 29.3% reduction. Increasing the thickness to 60 cm further reduced total electricity consumption to 1,476.1 kWh and operational CO₂ emissions to 894.5 kg, corresponding to an additional 1.9%-point reduction in annual electricity consumption and operational CO_2_ emissions compared with the 25 cm variant (Fig. 8a and c).
- **Spatial-performance trade-offs**: The results suggest that, while 60 cm masonry provides only marginal additional performance gains, it also results in increased wall thickness. For residential retrofitting, the 15–25 cm masonry variants offer a smaller spatial footprint while capturing a substantial portion of the observed thermal and environmental benefits. These configurations show clear improvements over baseline performance without introducing the constructability and spatial constraints associated with maximum-thickness scenarios.
- **Ultra-thin interventions**: The study evaluated a 6 cm date palm midrib panel (DPM. Pan.) as a thin bio-based cladding intervention, achieving a 21.9% reduction in discomfort hours (Fig. 9a) and 28.7% reduction in total heating and cooling electricity consumption (Fig. 9b). Compared with the 2 cm date palm plaster (DP. Pl.), the 6 cm cladding further reduced discomfort hours and total electricity consumption by 5.4 and 8.8% points, respectively. Although the 6 cm cladding showed better thermal performance, both bio-based interventions reduced overall electricity consumption relative to the evaluated high-mass masonry configurations. These findings suggest that thin bio-based cladding systems may provide a space-efficient alternative for selected retrofit applications.

### Comparative performance across worst-case orientations

The East and West orientations were selected for targeted evaluation of the waste-based masonry, based on their higher thermal loads identified in the Level 1 analysis.

- The East orientation, identified as the most critical for thermal discomfort and cooling electricity consumption, was evaluated under both NV and MMV modes to assess the thermal performance of the selected interventions in improving passive comfort and reducing active electricity consumption.
- The West orientation, identified as the least efficient due to its highest combined annual heating and cooling electricity consumption, was evaluated exclusively under MMV mode to assess the operational thermal performance of the selected interventions under thermally demanding conditions.

The study assessed the best-performing variants from each material category, as identified in the South orientation analysis. To examine performance variation across thickness extremes under thermally demanding conditions, simulations were conducted using the minimum (15 cm) and maximum (60 cm) thicknesses of the selected materials. These configurations were then benchmarked against the Level 2 scenarios, specifically X-C (Code Compliance) and X-BP (Best Practice), to compare waste by-product-based material systems with conventional insulation strategies.

#### Thermal performance in east orientation

**Fig. 10:**
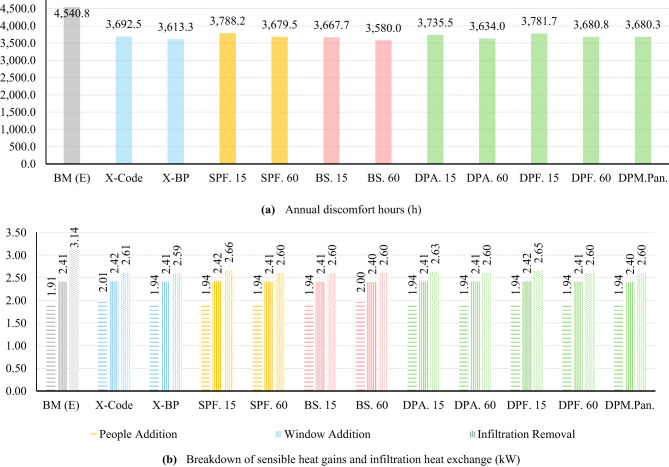
Comparative performance of the selected intervention scenarios under NV mode, relative to the BM for the East orientation. Numeric labels denote material thicknesses in centimeters.

**Fig. 11:**
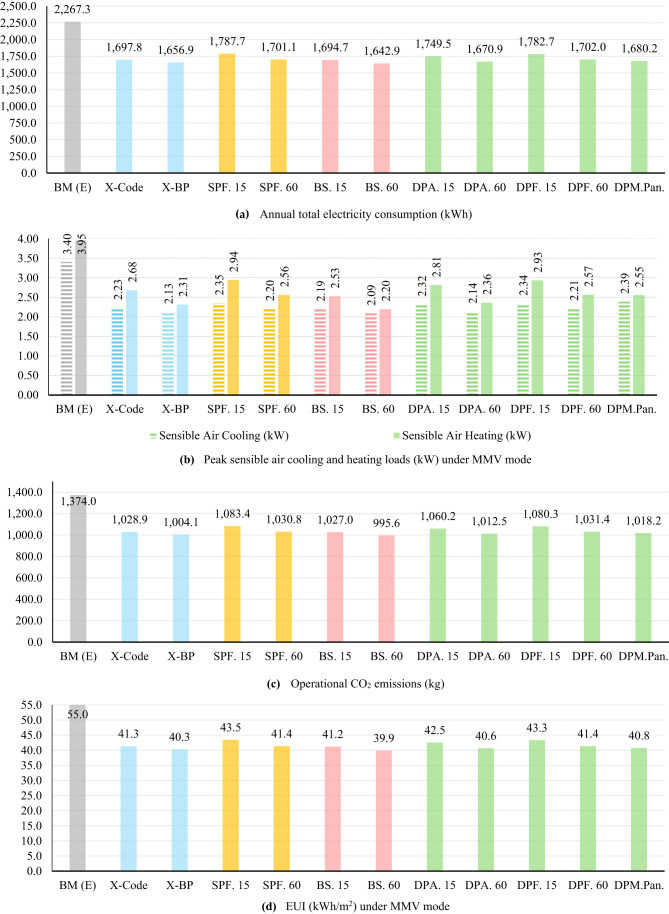
Comparative performance of the selected intervention scenarios for the East orientation under MMV mode. Numeric labels denote material thicknesses in centimeters.

**Fig. 12:**
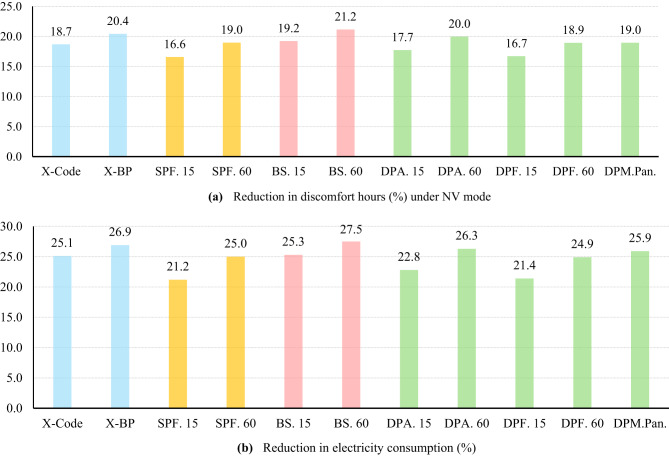
Comparative percentage improvements in thermal and energy performance of the selected intervention scenarios for the East orientation under NV and MMV modes. Numeric labels denote material thicknesses in centimeters.

Figures 10, 11, and 12 present simulation results for the East orientation under both NV and MMV modes. The following section examines the thermal performance of the selected scenarios, quantifying their effects on annual discomfort hours and peak sensible air-cooling loads relative to the BM and conventional benchmark scenarios.

- **NV mode and thermal comfort**: Analysis presented in Fig. 10a reveals clear performance differences across the evaluated scenarios. The BM recorded 4,540.8 annual discomfort hours. Among the interventions, the 60 cm BS variant achieved the largest reduction, lowering discomfort to 3,580.0 h. This corresponds to a 21.2% reduction (approximately 961 h), slightly exceeding the reduction observed in the X-BP benchmark scenario (20.4%). Furthermore, the 15 cm BS and 6 cm DPM Pan. Configurations achieved comparable performance, with discomfort reductions of 19.2% and 19.0%, respectively, as shown in Fig. 12a.
- **Thermal loads and heat gains**: Fig. 10b indicates that heat loss attributed to infiltration decreased from 3.14 kW in the BM to approximately 2.6 kW across all material configurations, with minimal variation among the evaluated variants. In contrast, internal heat gains from occupancy and solar gains through glazing remained consistent across all simulated scenarios, at approximately 1.9 kW and 2.4 kW, respectively.
- **MMV mode and energy efficiency**: Implementing these assemblies within MMV mode resulted in a consistent reduction in annual energy consumption. Total electricity consumption decreased from 2,267.3 kWh in the BM to 1,642.9 kWh for the 60 cm BS variant, representing a 27.5% reduction (Figs. 11a and 12b). As a result, the EUI declined from 55 kWh/m^2^ to 39.9 kWh/m^2^ (Fig. 11d), corresponding to a 27.5% reduction in operational CO₂ emissions.

#### Thermal performance in west orientation

The West orientation was evaluated under MMV mode to assess operational electricity performance under high thermal load conditions. Simulation results indicate that the BM West recorded a total electricity consumption of 2,280.5 kWh.

**Fig. 13:**
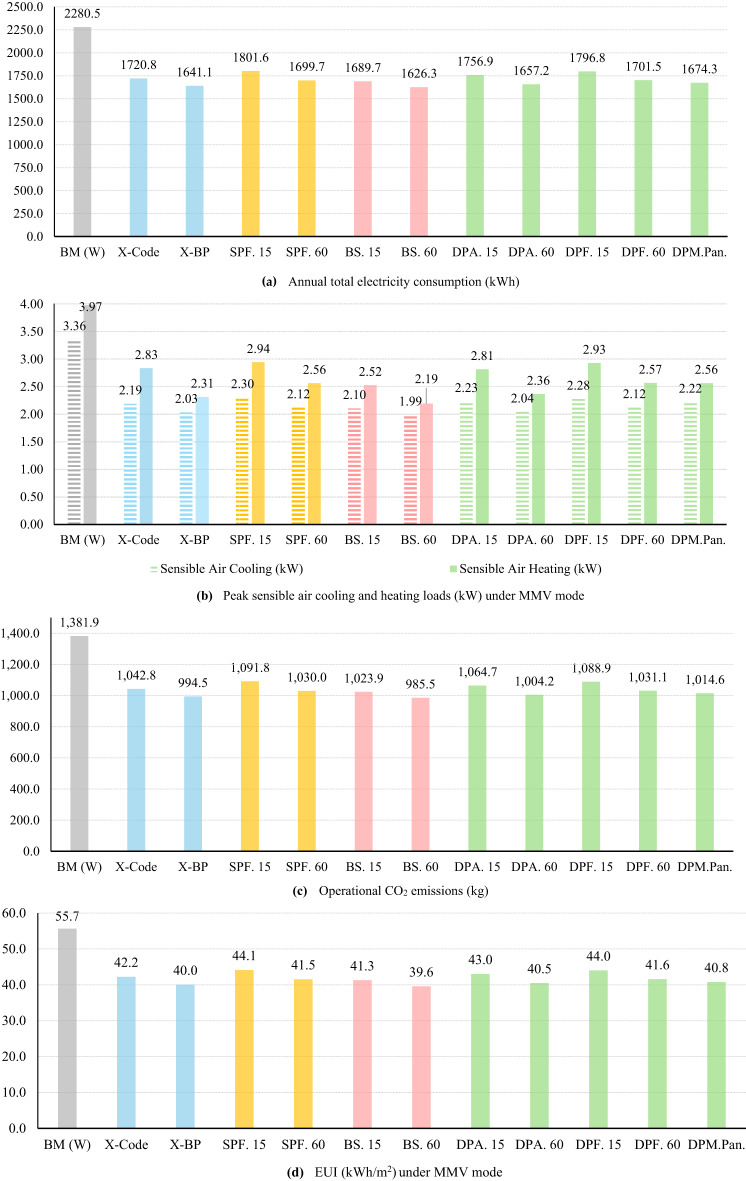
Comparative performance of the selected intervention scenarios under MMV mode for the West orientation. Numeric labels denote material thicknesses in centimeters.

**Fig. 14:**
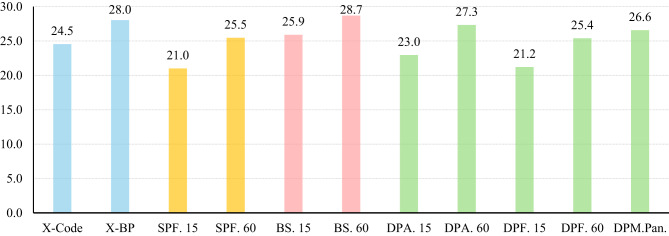
Comparative percentage reductions in annual electricity consumption (%) of the selected intervention scenarios for the West orientation under MMV mode. Numeric labels denote material thicknesses in centimeters.

- **Comparative efficiency**: Analysis of the retrofit scenarios indicates a reduction in annual electricity consumption across the selected assemblies (Fig. 13a). The synthetic benchmark (X-BP) recorded a consumption of 1,641.1 kWh, representing a 28% reduction relative to the BM. In comparison, the 15 cm and 60 cm bio-based BS configurations recorded 1,689.7 kWh and 1,626.3 kWh, corresponding to reductions of 25.9% and 28.7%, respectively (Fig. 14).
- **EUI and peak shaving**: Correspondingly, the EUI decreased from 55.7 kWh/m^2^ in the BM to 39.6 kWh/m^2^ in the BS 60 cm scenario (Fig. 13d). The lowest peak sensible air-cooling load was recorded for the BS 60 cm configuration (1.99 kW), representing a 40.8% reduction relative to the BM, while the other evaluated scenarios remained within a relatively narrow range of 2.0–2.3 kW (Fig. 13c). This corresponds to peak sensible air-cooling load reductions ranging from approximately 32.4% to 41.2% compared with the BM, indicating that waste-based masonry alternatives achieved performance comparable to synthetic insulation in the least efficient orientation identified in the Level 1 analysis.
- **Performance–space trade-offs**: The results indicate that both thin synthetic insulation and thicker waste-based masonry achieved substantial peak-shaving benefits. The 15 cm BS configuration represents an intermediate retrofit option, offering thermal performance comparable to the industrial benchmark while requiring less wall thickness than the 60 cm BS variant. These findings suggest that material thickness may be selected based on site-specific spatial and structural constraints while still achieving substantial reductions in peak sensible air-cooling loads.
- **Environmental impact**: Annual operational CO_2_ emissions decreased from 1,381.9 kg in the BM to 985.5 kg for the BS 60 cm variant and 1,023.9 kg for the BS 15 cm variant (Fig. 13c). These values represent total emission reductions of 28.7% and approximately 25.9%, respectively. The results demonstrate a consistent reduction in operational CO_2_ emissions across both waste-based thicknesses compared to the BM.

### Multi-orientation performance synthesis

Level 1 established the baseline thermal and energy performance of the BM across the four cardinal orientations under NV and MMV conditions. The results indicated overall thermal inadequacy, with all orientations remaining outside comfort conditions for approximately half of the annual hours under NV mode. Under MMV operation, orientation-dependent differences emerged, with the South orientation showing the most favorable overall energy performance, while the East and West orientations represented the most thermally demanding cases due to higher cooling and combined heating–cooling electricity consumption, respectively. Benchmarking against the RBEE Code further confirmed that the baseline envelope did not fully comply with the required thermal resistance standards, particularly for conditioned buildings and East/West facades. These findings established the analytical framework for the subsequent levels, with the South orientation selected for comparative evaluation all material configurations and the East and West orientations used as critical performance assessment cases.

Level 2 showed that conventional XPS retrofitting improved both passive comfort and active energy performance in the South orientation relative to the baseline model. Under NV conditions, all insulation strategies reduced annual discomfort hours, while under MMV operation, conventional insulation reduced electricity consumption, EUI, operational CO_2_ emissions, opaque surface conduction heat gains, and peak sensible thermal loads. The X-C code-compliant scenario achieved performance comparable to the X-BP configuration, indicating that compliance with current RBEE thermal standards provides a practical benchmark, while additional insulation thickness yielded incremental gains.

Level 3 indicated that waste and by-product-based retrofitting strategies improved both passive and active thermal performance in the South orientation, with several configurations showing performance comparable to conventional synthetic benchmarks. Among the evaluated options, the BS. CE-brick showed the strongest thermal performance, although gains beyond the 15–25 cm range were incremental and were offset by associated spatial implications. The 6 cm DPM panel also showed that a thinner bio-based cladding intervention can achieve competitive reductions in discomfort hours, electricity consumption, and operational CO_2_ emissions while preserving usable space.

A comparative evaluation of the East and West orientations showed that waste by-product-based masonry systems maintained competitive performance under the less favorable thermal exposure conditions identified in Level 1. In the East orientation, the BS 60 cm configuration achieved the largest reduction in annual discomfort hours, while moderate-thickness BS masonry and the DPM panel showed performance comparable to the conventional synthetic benchmark. Under MMV operation, both East and West analyses showed reductions in electricity consumption, EUI, and peak sensible air-cooling loads relative to the BM. In the West orientation, waste-based configurations achieved peak load reductions comparable to synthetic insulation, indicating that these material systems remained effective beyond the best-performing South orientation.

## Discussion

Simulation results show a significant gap between current construction and local energy demands. Southern orientation, recommended in Egyptian guidelines, provided the best thermal performance for the baseline model (BM). However, its energy use intensity (EUI) of 52.4 kWh/m^2^ is still more than twice the benchmark of 11–22.4 kWh/m^2^ for Egypt’s North Coast (Attia et al., 2012)^16^. This difference may reflect recent rises in ambient temperatures, which affect cooling needs. Cooling demand accounts for a large share of household electricity use in standard envelopes, highlighting their limits. The results also suggest that shading-only strategies, such as following RBEE external shading requirements, may not ensure adequate thermal performance. Improving thermal performance likely depends on both geometric shading and thermal properties of materials.

Level 2 interventions for the South orientation indicate that wall insulation was a major contributing factor to energy savings under MMV mode, accounting for a 42.9% reduction in opaque surface conduction loads in the code-compliance scenario. A 6 cm XPS thickness, used as a practical benchmark, aligns with established research as an effective insulation threshold. The observed 29.8% reduction in EUI, resulting from the transition from the BM to the best-practice configuration, reinforces the role of envelope performance in residential energy-efficiency strategies. The 3.3%-point energy performance gap suggests that, while standard compliance establishes a functional baseline, there remains measurable potential for further passive improvements. This finding indicates that increasing insulation thickness may help reduce the remaining performance gap and improve internal thermal stability.

A key finding of this study (Level 3) is that local waste by-product-based materials can achieve thermal performance comparable to synthetic counterparts for the South orientation, with selected configurations showing marginally higher electricity savings. BS masonry, at a 15 cm thickness, recorded a 27.9% reduction in annual electricity consumption, compared with the 26.7% reduction observed for the code-compliant synthetic benchmark (X-C scenario). These results suggest that, for Egypt’s North Coast climate, high-performing bio-based assemblies such as BS and the DPM panel, which achieved 28.8% electricity savings, may help mitigate cooling loads without requiring the larger thicknesses associated with high-mass alternatives. While thicknesses up to 60 cm were modeled to explore upper performance scenarios, the data indicate that the 15–25 cm range for BS captures most of the observed performance gains. This range appears to offer a practical balance between thermal lag and constructability, addressing spatial constraints while preserving much of the observed energy benefit.

The simulation findings suggest an underlying thermal mechanism that may explain this performance, particularly in the East orientation, where the building envelope appeared to moderate heat transfer between outdoor conditions and indoor thermal loads. Under NV mode, the 60 cm BS configuration showed a 17.2% reduction in infiltration-related heat exchange relative to the BM, comparable to that of high-performance synthetic insulation. Under MMV mode, the maximum peak sensible air-cooling load decreased to 2.1 kW for the 60 cm BS assembly. The 15 cm BS and DPM panel variants also showed comparable performance, maintaining peak sensible air-cooling loads within a narrow range despite their reduced thickness. Correspondingly, the 60 cm BS configuration was associated with a 27.5% reduction in EUI. Although windows remained a consistent source of residual heat gain (averaging 2.40 kW), similar to the BM, the waste-based materials were associated with reduced infiltration-related sensible heat exchange under high-solar-exposure conditions. Similarly, the West orientation results suggest that waste-based masonry may help mitigate late-day solar gains. The reduction in annual electricity consumption from 2,280.5 kWh to 1,626.3 kWh in the 60 cm BS scenario suggests that biocomposites can achieve performance comparable to synthetic insulation in selected metrics. This is reflected in a 40.8% reduction in maximum peak sensible air-cooling load for the bio-based masonry, compared with the 39.4% reduction observed for the 6 cm XPS layer (X-BP scenario). This performance difference may be associated with the material’s higher thermal mass, which appears to reduce the transmission of afternoon solar heat gains. Consequently, while synthetic insulation offers a more compact alternative, bio-based masonry may represent a practical thermal retrofit option in cases where spatial constraints permit increased envelope thickness, with potential benefits for peak-load reduction and thermal moderation.

Within this context, the 6 cm DPM panel may represent a practical option for urban retrofitting projects where preserving internal floor area is essential. Its 28.8% reduction in annual electricity consumption in the South orientation, without the substantial spatial footprint of masonry, suggests a viable approach for upgrading high-density residential units, particularly in the electricity-demanding West orientation (26.6% savings). Architecturally, this thin-profile assembly helps preserve usable internal space and may address external site constraints, such as narrow setbacks, where increasing envelope thickness is not feasible. However, its effectiveness appeared slightly reduced in the East orientation, with electricity savings decreasing by 0.7% points compared with the West orientation (25.9% vs. 26.6%). Under East-facing solar exposure conditions, high-mass bio-based configurations showed better thermal performance.

Although the discussion emphasizes the highest-performing and most space-efficient configurations, intermediate material variants also generally show performance improvements over the baseline. These options may offer greater flexibility for architectural applications, allowing trade-offs between moderate thermal gains, material availability, and practical implementation constraints to be considered. This diversity suggests that waste-based materials may be adaptable to a range of project requirements within the Egyptian context.

At a broader scale, the observed reduction in peak sensible air-cooling demand (22.3%–47% for the South orientation) may have implications for building services design. Such reductions could support smaller HVAC systems, thereby reducing initial costs and reducing peak electricity demand. The findings suggest that waste-based materials may represent a technically viable approach for improving thermal performance in Egyptian housing applications. By showing performance comparable to conventional insulation in selected scenarios, these materials may also support broader circular-economy objectives in the residential sector.

### Study scope and limitations

This study presents a simulation-based comparative assessment for waste-based retrofitting; however, several limitations must be acknowledged:

- **Simulation-based methodology and model validation**: This study represents a simulation-based comparative assessment rather than a calibrated predictive model. Owing to the absence of onsite monitored performance data for the selected housing typology, the DEM could not be validated against measured operational conditions. Consequently, the findings should be interpreted as comparative scenario-based performance trends rather than absolute predictions of real-world energy use. In addition, incomplete thermophysical datasets for certain waste-based composites limited full characterization of dynamic thermal storage behavior, as simulations relied primarily on literature-reported thermal conductivity values. Future experimental measurements and field calibration are required to strengthen predictive reliability.
- **Constructability and spatial efficiency**: Although a 60 cm “highest-performing thickness” was identified, this thickness introduces significant structural dead loads and urban setback constraints, such as building lines. To balance thermal performance with practical feasibility, the study prioritizes applications with high-performance thin layers (e.g., a 6 cm DPM panel) and moderate thicknesses (15–25 cm) for favorable orientations. This consideration highlights the need to balance thermal performance with structural and regulatory constraints in contemporary construction.
- **Material standardization and resilience**: The variability of agricultural by-products highlights the need for further research to standardize densities and thermal properties across manufacturing processes. Additionally, aspects such as moisture migration, mould resistance, and fire hazards were beyond the current scope and require experimental evaluation, particularly for coastal environments.
- **Economic and life cycle scope**: The present study focuses exclusively on operational thermal performance. For a comprehensive assessment, future research should incorporate Life Cycle Assessment (LCA) to evaluate cradle-to-grave impacts, as well as cost-benefit analyses to balance energy savings with initial investment and the deployment feasibility of locally sourced waste by-product-based materials.
- **Typological and scale constraints**: The results are based on a standalone residential prototype representing a high-exposure scenario. Performance outcomes may vary in high-density urban environments, where mutual overshadowing and differences in surface-to-volume ratios influence energy dynamics. Therefore, the findings should be interpreted within the climatic, typological, and urban context of the selected North Sinai case study.

## Conclusions

This multi-level thermal and energy analysis indicates a gap between mandatory code compliance and actual thermal performance. The study’s findings are summarized as follows:

- **Discrepancy between code compliance and comfort**: The analysis suggests that adherence to the Egyptian Energy Code (RBEE) does not necessarily ensure thermal comfort. Even with standard insulation (XPS) and legal shading requirements in place, significant discomfort hours persisted. This indicates that current regulatory benchmarks may not fully address thermal comfort in this case-study context, as they do not account for the cumulative effects of high thermal conductivity in high-exposure social housing prototypes.
- **Orientation-specific vulnerabilities**: The analysis identified a thermal hierarchy across the building orientations. The South orientation exhibited the most favorable thermal performance, whereas the Western orientation showed high thermal sensitivity due to intense late-day solar gains. In contrast, bio-based interventions in the East were associated with reductions of up to 961 annual discomfort hours under natural ventilation (NV), indicating that material-specific retrofitting may help moderate thermal stress in these orientations.
- **Ventilation-dependent strategies**: Orientation strategies may need to be aligned with the ventilation mode (NV versus MMV). While some orientations perform better in natural ventilation mode, others, particularly the West, may benefit from higher-resistance bio-insulation to reduce mechanical cooling demand. These findings suggest that building form and material selection should be considered as an integrated system rather than as isolated variables.
- **Performance of bio-based scenarios**: In critical orientations, bio-based configurations such as BS and DPM exhibited thermal performance comparable to synthetic benchmarks, particularly in selected peak thermal load metrics. The thermal mass of these local biomaterials may contribute to thermal buffering, helping reduce the impact of external solar fluctuations.
- **Efficiency versus spatial constraints**: Compared with the BS variant, the 6 cm DPM panel may provide a practical retrofit option for space-constrained residential units. Its slim profile may help balance energy-efficiency objectives with spatial limitations.
- **Environmental performance and retrofit potential**: The observed 28.8% reduction in operational CO₂ associated with the 6 cm DPM panel in the South orientation suggests potential relevance for similar residential retrofit applications in hot coastal climates. This approach illustrates how regional agricultural waste may be repurposed as a functional building component with potential thermal and environmental benefits.

Overall, this study suggests that an integrated approach combining orientation-sensitive design, envelope insulation, and locally sourced biomaterials may help reduce the building energy performance gap. These findings provide a comparative basis for improving thermal comfort and informing sustainable housing retrofit strategies in similar Mediterranean coastal contexts.

## Recommendations

Based on the research findings, the following recommendations are proposed to address the gap between existing building codes and enhanced thermal performance in social housing contexts.

- **Transition toward dynamic performance standards**: Regulatory authorities may consider evolving the RBEE framework from static minimum R-value compliance toward performance-based criteria. Such an approach could better reflect operational energy performance and more effectively account for the role of thermal mass under Egyptian climatic conditions.
- **Material standardization for broader implementation**: Transitioning from simulation-based proof-of-concept studies toward broader implementation may require standardized manufacturing protocols for bio-based materials. Future research should prioritize the characterization and standardization of density, thermal conductivity, and related thermophysical properties across agricultural waste batches to support more consistent performance assessment and alignment with future certification standards.
- **Context-aware envelope strategies**: To address spatial and constructability constraints, a tiered design approach may be considered:
- **External space availability**: High-thermal-mass masonry (such as BS CE-brick assemblies) may be appropriate where sufficient external space allows thicker envelope retrofits to enhance passive thermal buffering.
- **Space-constrained urban areas**: Hybrid systems incorporating thin bio-based insulation panels (such as DPM) may offer an alternative for improving thermal performance while minimizing spatial impact on internal floor area.
- **Orientation-specific design guidelines**: Building design frameworks may consider incorporating orientation-sensitive thermal resistance strategies, particularly for East and West facades, which were associated with higher thermal loads in the present case study. Additional solar-gain mitigation strategies may be beneficial under such conditions. Future revisions of the RBEE Code may also consider incorporating orientation-dependent thermal resistance requirements or performance-based compliance pathways for high-exposure facades.
- **Supporting circular economy integration**: To facilitate broader adoption of industrial and agricultural waste by-products in construction, policymakers may consider technical support mechanisms, pilot implementation programs, or policy incentives aligned with circular economy objectives. Further assessment is required to evaluate long-term economic feasibility.
- **Minimally invasive retrofitting approaches**: For existing building stock, thin bio-based insulation systems may represent practical retrofit options where structural, spatial, or architectural constraints limit extensive envelope modifications.
- **Integrated design and future research**: As this study is based on comparative simulation analysis, further research incorporating Life Cycle Assessment (LCA), cost-benefit analysis, material prototyping, and experimental validation is needed to evaluate the long-term thermal, economic, and structural performance of these interventions under real-world operating conditions.

## Supplementary Information

Below is the link to the electronic supplementary material.

Supplementary Material 1

## Data Availability

All datasets generated and analysed during the current study are included within the article. No copyrighted or restricted data have been shared.

## Abbreviations

BM: Baseline model
BS: Barley straw
CE-Brick: Compressed earth brick
CO_2_: Carbon dioxide
DB: DesignBuilder
DEM: Dynamic energy modeling
DP: Date palm
DPA: Date palm ash
DPF: Date palm fiber
DPM: Date palm midrib
E-Brick: Earth brick
EPS: Expanded polystyrene
EUI: Energy use intensity
MMV: Mixed-mode ventilation
NV: Natural ventilation
PF: Polystyrene foam
RBEE: Residential building energy efficiency code
S: Alkali-activated ground granulated blast furnace slag
SGR: Shaded glass ratio
SHGC: Solar heat gain coefficient
TMYx: Extended typical meteorological year
X-BP: Best-practice XPS scenario
X-C: Code-compliant XPS scenario
X-H: Homogeneous XPS scenario
XPS: Extruded polystyrene
