# Supplementary material for: Evaluating waste based retrofit strategies for thermal performance improvement in North Sinai social housing
Source: Sci Rep. 2026 Aug 12;16:25101. doi: 10.1038/s41598-026-64729-9 (PMC13469632; doi:10.1038/s41598-026-64729-9)
Supplement: Supplementary file 1 [file 41598_2026_64729_MOESM1_ESM.docx]

Table A1: Performance comparison of the BM across the four cardinal orientations under NV mode

| **Orientation** | | **Wall**  **R-Value**  **(m^2^** ·**K/W)** | **Annual Discomfort Hours (ASHRAE 55, 80% Acceptability)** | **Sensible heat gain components (W)** | | |
| --- | --- | --- | --- | --- | --- | --- |
|  |  |  |  | People Addition | Window Addition | Infiltration Removal |
|  | North  (0°) | 0.573  (0.29 m thickness) | 4,432.00 | 1,923.91 | 1,868.49 | 3,115.59 |
|  | East  (90°) |  | 4,540.83 | 1,912.12 | 2,409.25 | 3,142.56 |
|  | South  (180°) |  | 4,408.5 | 1,933.50 | 1,771.51 | 2,998.49 |
|  | West  (270°) |  | 4,478.00 | 1,913.22 | 2,351.71 | 3,158.34 |

Table A2: Performance comparison of the BM across the four cardinal orientations under MMV mode

| **Orientation** | | **Annual electricity consumption (kWh)** | | | **Operational**  **CO_2_**  **emissions**  **(kg)** | **EUI (kWh/m^2^)** | **Peak Loads** (W) | | **Opaque surface conduction and other heat gain** | |
| --- | --- | --- | --- | --- | --- | --- | --- | --- | --- | --- |
|  |  | Cooling | Heating | Total |  |  | Sensible Air Cooling | Sensible Air Heating | Addition | Removal |
|  | North  (0°) | 1,941.15 | 244.70 | 2,185.85 | 1,324.62 | 53.17 | 3,452.39 | 3,951.32 | 3,450.98 | 3,861.20 |
|  | East  (90°) | 2,034.69 | 232.58 | 2,267.27 | 1,373.96 | 55.00 | 3,399.85 | 3,952.94 | 3,391.69 | 3,929.44 |
|  | South  (180°) | 1,892.80 | 256.65 | 2,149.45 | 1,302.57 | 52.40 | 3,442.76 | 3,959.12 | 3,433.94 | 3,826.41 |
|  | West  (270°) | 2,000.5 | 280.01 | 2,280.51 | 1,381.99 | 55.65 | 3,355.49 | 3,972.40 | 3,349.61 | 3,983.05 |

Table A3: Performance comparison of the BM at the South orientation across all intervention scenarios under NV mode

| **Orientation** |  | **Wall Thickness**  **(m)** | **Wall**  **R-Value**  **(m^2^** ·**K/W)** | **Annual Discomfort Hours (ASHRAE 55, 80% Acceptability)** | **Sensible heat gain components (W)*** | | |
| --- | --- | --- | --- | --- | --- | --- | --- |
|  |  |  |  |  |  |  |  |
|  |  |  |  |  | People Addition | Window Addition | Infiltration Removal |
|  | BM South (180°) | 0.29 | 0.573 | 4,408.5 | 1,933.502 | 1,771.506 | 2,998.49 |
| XPS | X-C | 0.31 | 1.287 | 3,361.83 | 1,956.971 | 1,789.147 | 2,417.86 |
|  | X-H |  |  | 3,390 | 1,956.428 | 1,781.950 | 2,395.11 |
|  | X-BP | 0.35 | 2.716 | 3,282.34 | 1,967.312 | 1,780.258 | 2,376.35 |
| Byproduct Industrial in E-Brick | CE. 0.15 m | 0.44 | 0.692 | 3,639.67 | 1,961.112 | 1,784.239 | 2,509.39 |
|  | CE. 0.25 m | 0.54 | 0.771 | 3,592.17 | 1,962.410 | 1,783.149 | 2,482.35 |
|  | CE. 0.40 m | 0.69 | 0.890 | 3,522.16 | 1,964.405 | 1,783.185 | 2,448.79 |
|  | CE. 0.60 m | 0.89 | 1.048 | 3,461 | 1,965.298 | 1,782.517 | 2,426.06 |
|  | S. CE 0.15 m | 0.44 | 0.726 | 3,617.16 | 1,961.725 | 1,783.660 | 2,495.94 |
|  | S. CE 0.25 m | 0.54 | 0.829 | 3,554.84 | 1,963.792 | 1,783.629 | 2,460.21 |
|  | S. CE 0.40 m | 0.69 | 0.982 | 3,485.16 | 1,964.981 | 1,782.805 | 2,433.34 |
|  | S. CE 0.60 m | 0.89 | 1.187 | 3,421.67 | 1,965.871 | 1,782.048 | 2,414.36 |
|  | SPF. CE 0.15 m | 0.44 | 0.788 | 3,583.67 | 1,962.674 | 1,782.990 | 2,478.62 |
|  | SPF. CE 0.25 m | 0.54 | 0.931 | 3,503.83 | 1,964.641 | 1,783.033 | 2,440.75 |
|  | SPF. CE 0.40 m | 0.69 | 1.146 | 3,432.83 | 1,965.763 | 1,782.189 | 2,418.61 |
|  | SPF. CE 0.60 m | 0.89 | 1.432 | 3,381.5 | 1,966.435 | 1,781.472 | 2,400.19 |
| Barely Straw | BS. 0.15 m | 0.44 | 1.554 | 3,365.67 | 1,966.542 | 1,781.266 | 2,395.11 |
|  | BS. 0.25 m | 0.54 | 2.186 | 3,315.83 | 1,967.095 | 1,780.681 | 2,381.08 |
|  | BS. 0.40 m | 0.69 | 3.153 | 3,262.83 | 1,967.307 | 1,779.969 | 2,371.97 |
|  | BS. 0.60 m | 0.89 | 4.444 | 3,237.5 | 1,967.333 | 1,779.412 | 2,364.34 |
| Date Palm By-Products | DP. Pl 0.02 m E. | 0.31 | 0.623 | 3,680.67 | 1,959.287 | 1,783.898 | 2,541.40 |
|  | DP. Pl 0.04 m E. | 0.33 | 0.674 | 3,645.67 | 1,960.532 | 1,780.691 | 2,516.21 |
|  | DP. Pl 0.02 m I. | 0.31 | 0.623 | 3,675.5 | 1,959.098 | 1,784.063 | 2,540.20 |
|  | DP. Pl 0.04 m I. | 0.33 | 0.674 | 3,649.5 | 1,959.924 | 1,780.463 | 2,517.69 |
|  | DPA. 0.15 m | 0.44 | 0.941 | 3,468.83 | 1,963.792 | 1,772.900 | 2,444.28 |
|  | DPA. 0.25 m | 0.54 | 1.172 | 3,415.5 | 1,964.690 | 1,773.888 | 2,425.44 |
|  | DPA. 0.40 m | 0.69 | 1.517 | 3,352.84 | 1,965.678 | 1,776.234 | 2,410.19 |
|  | DPA. 0.60 m | 0.89 | 1.978 | 3,298.5 | 1,966.807 | 1,777.537 | 2,389.74 |
|  | DPF. 0.15 m | 0.44 | 0.801 | 3,575 | 1,963.046 | 1,783.050 | 2,472.66 |
|  | DPF. 0.25 m | 0.54 | 0.938 | 3,504.17 | 1,964.213 | 1,782.251 | 2,444.73 |
|  | DPF. 0.40 m | 0.69 | 1.143 | 3,435.51 | 1,965.453 | 1,781.552 | 2,423.56 |
|  | DPF. 0.60 m | 0.89 | 1.417 | 3,385.33 | 1,966.099 | 1,781.015 | 2,402.83 |
|  | DPM. Pan | 0.35 | 1.434 | 3,441.84 | 1,960.055 | 1,770.691 | 2,395.04 |
| *The terms “People Addition,” “Window Addition,” and “Infiltration Removal” are software-generated output labels referring to occupant-sensible gains, glazing-related gains, and infiltration-related heat exchange, respectively.  (X-C) Applied to non-compliant components in accordance with the RBEE Code for unconditioned buildings (East and West facades for the BM under NV mode).  (X-H) A uniform 20 mm insulation was applied to all facades (North, East, South, and West).  (X-BP) A uniform 60 mm insulation was applied to all facades and the roof.  DP. Pl. = Date Palm Plaster; E = external application; I = internal application; numeric values indicate material thickness in meters (0.02 and 0.04 m). | | | | | | | |

Table A4: Performance comparison of the BM at the South orientation across all intervention scenarios under MMV mode

| **Orientation**   | | |  | **Annual electricity consumption (kWh)** | | | **Operational CO_2_**  **emissions**  **(kg)** | **EUI**  **(kWh**  **/m^2^)** | | **Peak loads**  **(W)** | | **Opaque surface conduction and other heat addition** | |
| --- | --- | --- | --- | --- | --- | --- | --- | --- | --- | --- | --- | --- | --- |
|  |  |  |  |  |  |  |  |  |  |  |  |  |  |
|  |  |  | Cooling | | Heating | Total |  |  |  | Sensible Air Cooling | Sensible Air Heating | Addition | Removal |
|  | | BM South (180°) | 1,892.8 | | 256.65 | 2,149.45 | 1,302.57 | 52.40 | | 3442.76 | 3,959.12 | 3,433.94 | 3,826.41 |
| XPS | | X-C | 1,348.31 | | 227.29 | 1,575.6 | 954.81 | 38.69 | | 2,155.23 | 2,811.80 | 1,960.16 | 2,840.82 |
|  |  | X-H | 1,357.19 | | 204.76 | 1,561.95 | 946.54 | 38.21 | | 2,054.07 | 2,618.15 | 1,857.14 | 2,635.81 |
|  |  | X-BP | 1,309.67 | | 194.73 | 1,504.4 | 911.67 | 36.78 | | 1,869.68 | 2,311.87 | 1,669.31 | 2,320.35 |
| Byproduct Industrial in E-Brick | | CE. 0.15 m | 1,470.39 | | 221.03 | 1,691.42 | 1025 | 41.37 | | 2,539.68 | 3,055.20 | 2,343.49 | 2,915.61 |
|  |  | CE. 0.25 m | 1,443.61 | | 218.84 | 1,662.45 | 1007.44 | 40.67 | | 2,384.15 | 2,961.75 | 2,169.08 | 2,820.35 |
|  |  | CE. 0.40 m | 1,409.45 | | 215.99 | 1,625.44 | 985.02 | 39.78 | | 2,297.55 | 2,854.68 | 2,070.02 | 2,708.56 |
|  |  | CE. 0.60 m | 1,387.41 | | 211.94 | 1,599.35 | 969.2 | 39.14 | | 2,162.79 | 2,740.29 | 1,968.37 | 2,646.06 |
|  |  | S. CE 0.15 m | 1,456.37 | | 220 | 1,676.37 | 1015.88 | 41.01 | | 2,457.04 | 3,012.16 | 2,254.82 | 2,871.80 |
|  |  | S. CE 0.25 m | 1,423.88 | | 217.67 | 1,641.55 | 994.78 | 40.17 | | 2,308.85 | 2,904.46 | 2,120.50 | 2,761.14 |
|  |  | S. CE 0.40 m | 1,394.77 | | 213.58 | 1,608.35 | 974.66 | 39.36 | | 2,219.18 | 2,780.90 | 2,025.94 | 2,688.81 |
|  |  | S. CE 0.60 m | 1,373.45 | | 209.07 | 1,582.52 | 959 | 38.72 | | 2,102.40 | 2,664.62 | 1,906.36 | 2,567.82 |
|  |  | SPF. CE 0.15 m | 1,438.5 | | 218.33 | 1,656.83 | 1004.04 | 40.54 | | 2,355.15 | 2,944.10 | 2,138.21 | 2,802.00 |
|  |  | SPF. CE 0.25 m | 1,401.96 | | 214.87 | 1,616.83 | 979.8 | 39.57 | | 2,244.95 | 2,821.56 | 2,057.78 | 2,674.03 |
|  |  | SPF. CE 0.40 m | 1,377.81 | | 209.93 | 1,587.74 | 962.17 | 38.85 | | 2,125.80 | 2,686.50 | 1,933.42 | 2,590.03 |
|  |  | SPF. CE 0.60 m | 1,352.61 | | 205.08 | 1,557.69 | 943.96 | 38.11 | | 2,018.31 | 2,562.01 | 1,819.46 | 2,576.07 |
| BS CE-Brick | | BS. 0.15 m | 1,346.34 | | 203.58 | 1,549.92 | 939.25 | 37.92 | | 1,989.73 | 2,524.64 | 1,790.52 | 2,536.95 |
|  |  | BS. 0.25 m | 1,320.77 | | 197.69 | 1,518.46 | 920.18 | 37.13 | | 1,923.34 | 2,382.88 | 1,723.24 | 2,385.27 |
|  |  | BS. 0.40 m | 1,301.08 | | 192.95 | 1,494.03 | 905.38 | 36.52 | | 1,840.65 | 2,270.30 | 1,643.68 | 2,276.10 |
|  |  | BS. 0.60 m | 1,286.33 | | 189.72 | 1,476.05 | 894.48 | 36.08 | | 1,826.40 | 2,193.92 | 0.00 | 2,194.46 |
| Date Palm By-Products | | DP. Pl. 0.02 m E. | 1,495.4 | | 223.12 | 1,718.52 | 1,041.42 | 42.02 | | 2,674.83 | 3,152.34 | 2,481.57 | 3,011.84 |
|  |  | DP. Pl. 0.04 m E. | 1,471.98 | | 222.57 | 1,694.55 | 1,026.9 | 41.45 | | 2,542.18 | 3,078.89 | 2332.53 | 2,936.28 |
|  |  | DP. Pl. 0.02 m I. | 1,494.83 | | 221.38 | 1,716.21 | 1,040.02 | 41.95 | | 2,665.25 | 3,150.32 | 2477.05 | 3,011.87 |
|  |  | DP. Pl. 0.04 m I. | 1,471.76 | | 219.99 | 1,691.75 | 1,025.2 | | 41.37 | 2,565.78 | 3,077.01 | 2372.37 | 2,938.38 |
|  |  | DPA. 0.15 m | 1,403.12 | | 214.32 | 1,617.44 | 980.17 | | 39.58 | 2,181.32 | 2,814.27 | 1,973.45 | 2,665.52 |
|  |  | DPA. 0.25 m | 1,375.27 | | 208.13 | 1,583.4 | 959.54 | | 38.74 | 2,081.44 | 2,659.81 | 1,879.78 | 2,552.90 |
|  |  | DPA. 0.40 m | 1,347.01 | | 201.54 | 1,548.55 | 938.42 | | 37.87 | 1,946.97 | 2,512.95 | 1,750.87 | 2,389.12 |
|  |  | DPA. 0.60 m | 1,319.26 | | 198.03 | 1,517.29 | 919.47 | | 37.11 | 1,854.82 | 2,360.98 | 722.60 | 2,219.54 |
|  |  | DPF. 0.15 m | 1,432.27 | | 218.11 | 1,650.38 | 1,000.13 | | 40.38 | 2,338.74 | 2,930.86 | 2,151.60 | 2,788.15 |
|  |  | DPF. 0.25 m | 1,405.1 | | 214.33 | 1,619.43 | 981.37 | | 39.63 | 2,259.07 | 2,815.76 | 2,066.83 | 2,667.65 |
|  |  | DPF. 0.40 m | 1,380.51 | | 209.55 | 1,590.06 | 963.58 | | 38.90 | 2,153.37 | 2,687.47 | 1,957.45 | 2,590.81 |
|  |  | DPF. 0.60 m | 1,356.41 | | 205.04 | 1,561.45 | 946.24 | | 38.20 | 2,046.99 | 2,567.18 | 1,850.37 | 2,581.41 |
|  |  | DPM. Pan | 1,344.77 | | 186.6 | 1,531.37 | 928.01 | | 37.36 | 2,097.38 | 2,553.66 | 1,933.25 | 2,455.76 |
|  | (X-C) Applied to non-compliant components in accordance with the RBEE Code for conditioned buildings (East, West, and South facades for the BM under MMV mode).  (X-H) A uniform 20 mm insulation was applied to all facades (North, East, South, and West).  (X-BP) A uniform 60 mm insulation was applied to all facades and the roof.  DP. Pl = Date Palm Plaster; E = external application; I = internal application; Numeric values indicate material thickness in meters (0.02 and 0.04 m). | | | | | | | | | | | | |

Table A5: Performance comparison of the BM at the East orientation across the selected intervention scenarios under NV mode

| **Orientation**   | | **Wall thickness**  **(m)** | **Wall**  **R-Value (m^2^·K/W)** | **Annual Discomfort Hours (ASHRAE 55, 80% Acceptability)** | **Sensible heat gain components (W)*** | | |
| --- | --- | --- | --- | --- | --- | --- | --- |
|  |  |  |  |  | People  Addition | Window  Addition | Infiltration  Removal |
| BM East (90°) | | 0.29 | 0.566 | 4,540.83 | 1,912.12 | 2,409.25 | 3,142.56 |
| XPS | X-C^**^ | 0.31 | 1.281 | 3,692.50 | 2,007.67 | 2,424.42 | 2,613.65 |
|  | X- BP^***^ | 0.35 | 2.709 | 3,613.34 | 1,942.05 | 2,408.49 | 2,590.36 |
| CSEB | SPF. CE 0.15 m | 0.44 | 0.788 | 3,788.16 | 1,940.44 | 2,416.37 | 2,657.77 |
|  | SPF. CE 0.60 m | 0.89 | 1.426 | 3,679.50 | 1,942.65 | 2,411.97 | 2,598.66 |
| BS | BS. 0.15 m | 0.44 | 1.554 | 3,667.67 | 1,942.61 | 2,411.46 | 2,597.49 |
|  | BS. 0.60 m | 0.89 | 4.438 | 3,580.01 | 2,004.49 | 2,404.53 | 2,602.43 |
| DP | DPA. 0.15 m | 0.44 | 0.941 | 3,735.5 | 1,941.45 | 2,405.20 | 2,627.95 |
|  | DPA. 0.60 m | 0.89 | 1.972 | 3,634.00 | 1,941.93 | 2,407.10 | 2,596.17 |
|  | DPF. 0.15 m | 0.44 | 0.801 | 3,781.67 | 1,940.78 | 2,416.35 | 2,651.72 |
|  | DPF. 0.60 m | 0.89 | 1.412 | 3,680.83 | 1,942.45 | 2,411.56 | 2,603.72 |
|  | DPM. Pan. | 0.35 | 1.434 | 3,680.33 | 1,935.24 | 2,400.35 | 2,601.54 |
| *The terms “People Addition,” “Window Addition,” and “Infiltration Removal” are software-generated output labels referring to occupant-sensible gains, glazing-related gains, and infiltration-related heat exchange, respectively.  **X-C was applied to East and West facades (in accordance with RBEE requirements for unconditioned buildings).  ***X-BP was applied to all facades in accordance with best-practice thickness specifications. | | | | | | | |

Table A6: Performance comparison of the BM at the East orientation across the selected intervention scenarios under MMV mode

| **Orientation**   | | **Annual electricity consumption (kWh)** | | | **Operational**  **CO^2^ emissions (kg)** | **EUI**  **(kWh/m^2^)** | **Peak loads (W)** | |
| --- | --- | --- | --- | --- | --- | --- | --- | --- |
|  |  |  |  |  |  |  |  |  |
|  |  | Cooling | Heating | Total |  |  | Sensible  Air  Cooling | Sensible  Air  Heating |
|  |  |  |  |  |  |  |  |  |
| BM East (90°) | | 2,034.69 | 232.58 | 2,267.27 | 1,373.96 | 55.00 | 3,399.85 | 3,952.94 |
| XPS | X-C ^*^ | 1,501.67 | 196.16 | 1,697.83 | 1,028.88 | 41.34 | 2,230.53 | 2,678.58 |
|  | X- BP ^**^ | 1,472.08 | 184.81 | 1,656.89 | 1,004.07 | 40.30 | 2,125.98 | 2,312.94 |
| CSEB | SPF. CE 0.15 m | 1,592.28 | 195.44 | 1,787.72 | 1,083.36 | 43.45 | 2,347.96 | 2,944.48 |
|  | SPF. CE 0.60 m | 1,511.26 | 189.79 | 1,701.05 | 1,030.83 | 41.37 | 2,204.28 | 2,560.34 |
| BS | BS. 0.15 m | 1,505.30 | 189.35 | 1,694.65 | 1,026.96 | 41.22 | 2,191.51 | 2,525.78 |
|  | BS. 0.60 m | 1,467.95 | 174.91 | 1,642.86 | 995.57 | 39.90 | 2,085.50 | 2,195.00 |
| DP | DPA. 0.15 m | 1,555.03 | 194.48 | 1,749.51 | 1,060.20 | 42.54 | 2,318.21 | 2,813.81 |
|  | DPA. 0.60 m | 1,484.48 | 186.37 | 1,670.85 | 1,012.53 | 40.64 | 2,137.93 | 2,361.33 |
|  | DPF. 0.15 m | 1,587.27 | 195.44 | 1,782.71 | 1,080.32 | 43.33 | 2,340.16 | 2,931.37 |
|  | DPF. 0.60 m | 1,512.15 | 189.88 | 1,702.03 | 1,031.43 | 41.39 | 2,206.99 | 2,565.19 |
|  | DPM. Pan. | 1,507.70 | 172.47 | 1,680.17 | 1,018.18 | 40.76 | 2,386.47 | 2,553.42 |
| *X-C was applied to East, West and South facades (in accordance with RBEE requirements for air-conditioned buildings).  ** X-BP was applied to all facades in accordance with best-practice thickness specifications. | | | | | | | | |

Table A7: Performance comparison of the BM at the West orientation across the selected intervention scenarios under MMV mode

| **Orientation**   | | **Annual electricity consumption (kWh)** | | | **Operational**  **CO_2_ emissions (kg)** | **EUI (kWh/m^2^)** | **Peak loads (W)** | |
| --- | --- | --- | --- | --- | --- | --- | --- | --- |
|  |  | Cooling | Heating | Total |  |  | Sensible Air  Cooling | Sensible Air Heating |
|  |  |  |  |  |  |  |  |  |
| BM West (270°) | | 2,000.50 | 280.01 | 2,280.51 | 1,381.99 | 55.65 | 3,355.49 | 3,972.40 |
| XPS | X-C ^*^ | 1,476.25 | 244.57 | 1,720.82 | 1,042.81 | 42.23 | 2,189.61 | 2,831.69 |
|  | X- BP ^**^ | 1,441.52 | 199.6 | 1,641.12 | 994.52 | 40.03 | 2,033.50 | 2,311.17 |
| CSEB | SPF. CE 0.15 m | 1,560.75 | 240.83 | 1,801.58 | 1,091.75 | 44.10 | 2,301.23 | 2,939.85 |
|  | SPF. CE 0.60 m | 1,482.69 | 217.02 | 1,699.71 | 1,030.03 | 41.54 | 2,118.18 | 2,561.34 |
| BS | BS. 0.15 m | 1,475.17 | 214.52 | 1,689.69 | 1,023.95 | 41.28 | 2,104.41 | 2,524.45 |
|  | BS. 0.60 m | 1,442.24 | 184.07 | 1,626.31 | 985.54 | 39.57 | 1,986.05 | 2,193.40 |
| DP | DPA. 0.15 m | 1,523.63 | 233.31 | 1,756.94 | 1,064.71 | 43.00 | 2,231.30 | 2,811.87 |
|  | DPA. 0.60 m | 1,450.78 | 206.37 | 1,657.15 | 1,004.23 | 40.46 | 2,041.21 | 2,363.41 |
|  | DPF. 0.15 m | 1,556.47 | 240.35 | 1,796.82 | 1,088.87 | 43.99 | 2,275.00 | 2,926.78 |
|  | DPF. 0.60 m | 1,484.37 | 217.13 | 1,701.50 | 1,031.11 | 41.58 | 2,121.13 | 2,566.44 |
|  | DPM. Pan. | 1,477.67 | 196.62 | 1,674.29 | 1,014.62 | 40.79 | 2,219.39 | 2,563.25 |
| *X-C was applied to East, West, and South facades (in accordance with RBEE requirements for air-conditioned buildings).  **X-BP was applied to all facades in accordance with best-practice thickness specifications. | | | | | | | | |
